# A flexible parametric accelerated failure time model and the extension to time-dependent acceleration factors

**DOI:** 10.1093/biostatistics/kxac009

**Published:** 2022-05-26

**Authors:** Michael J Crowther, Patrick Royston, Mark Clements

**Affiliations:** Department of Medical Epidemiology and Biostatistics, Karolinska Institutet, Box 281, S-171 77 Stockholm, Sweden; MRC CTU at UCL, 90 High Holborn, Holborn, London WC1V 6LJ, UK; Department of Medical Epidemiology and Biostatistics, Karolinska Institutet, Box 281, S-171 77 Stockholm, Sweden

**Keywords:** Accelerated failure time, Causal inference, Software, Survival analysis, Time-dependent effects

## Abstract

Accelerated failure time (AFT) models are used widely in medical research, though to a much lesser extent than proportional hazards models. In an AFT model, the effect of covariates act to accelerate or decelerate the time to event of interest, that is, shorten or extend the time to event. Commonly used parametric AFT models are limited in the underlying shapes that they can capture. In this article, we propose a general parametric AFT model, and in particular concentrate on using restricted cubic splines to model the baseline to provide substantial flexibility. We then extend the model to accommodate time-dependent acceleration factors. Delayed entry is also allowed, and hence, time-dependent covariates. We evaluate the proposed model through simulation, showing substantial improvements compared to standard parametric AFT models. We also show analytically and through simulations that the AFT models are collapsible, suggesting that this model class will be well suited to causal inference. We illustrate the methods with a data set of patients with breast cancer. Finally, we provide highly efficient, user-friendly Stata, and R software packages.

## 1. Introduction

Accelerated failure time (AFT) models are commonly used in a variety of settings within the medical literature (Collett, 2003). The interpretation of an acceleration factor can be considered more intuitive, directly adjusting the survival time, either increasing or decreasing it, compared to the interpretation of a hazard ratio, meaning a relative increase or decrease in the event rate (Swindell, 2009). A parametric approach tends to be favored when fitting an AFT model; however, parametric models are limited by the flexibility of the distribution chosen (Cox *and others*, 2007; Cox, 2008). Parametric AFT models are particular prevalent in economic decision modeling, where it is emphasized to fit a wide variety of parametric models (either proportional hazards or AFT), to obtain the “best fitting” model (Latimer, 2013). Often, extrapolation is required to calculate survival across a lifetime horizon, and hence parametric and flexible approaches are needed. Of course, extrapolation is fraught with dangers, and arguably should only be attempted in the presence of appropriate external data. 

To our knowledge, the most flexible fully (standard) parametric AFT model is the generalized F distribution, a four parameter distribution described by Cox (2008), which often suffers from convergence problems. This contains the more widely used (due to availability of software) generalized gamma as a special case (Cox *and others*, 2007). Many authors have compared and contrasted AFT models with the more commonly used proportional hazards metric (Kay and Kinnersley, 2002; Orbe *and others*, 2002). Lambert *and others* (2004) developed a mixture AFT model with frailties, where a short term hazard component was modeled with a Gompertz distribution, and the long term hazard component could be any of the standard parametric AFT models. There have been several efforts to develop smooth AFT models, including mixtures of normal densities (Komárek *and others*, 2005), kernel smoothed densities (Zeng and Lin, 2007), and seminonparametric densities (Zhang and Davidian, 2008). A software implementation of the mixture of normal densities has received modest attention. Rubio *and others* (2019) recently proposed a general hazards-based model, utilizing the exponentiated-Weibull model to model the baseline function. Recently, Pang *and others* (2021) proposed a flexible B-spline approach to modeling the baseline function in an AFT framework, and also provided R code. We take a similar vein in this article, but provide a number of important developments. 

Within a proportional hazards metric, the Royston–Parmar flexible parametric model has grown in popularity in recent years, with a number of extensions and developments being proposed (Royston and Lambert, 2011; Liu *and others*, 2018). The fundamental strength of the model is to use restricted cubic splines to model the underlying baseline function (regardless of scale), and any time-dependent effects. However, there are known limitations with models based on hazard ratios, where the hazard ratios are not collapsible across covariates not associated with the exposure of interest (Martinussen and Vansteelandt, 2013), while AFTs are known to be robust to omitted covariates (Hougaard, 1999). Together, this motivates the incorporation of a flexible framework into an AFT paradigm. 

AFT models make the assumption of a constant acceleration factor, that is, the effect of a covariate remains the same across follow-up time, similar to the proportional hazards assumption. Clearly this assumption is open to violation. This motivates the relaxation of the constant acceleration factor to allow time-dependency, similarly to modeling of nonproportional hazards. This has been described within a generalized gamma AFT model by Cox *and others* (2007). In this article, we further relax the constant acceleration factor assumption, within the flexible parametric AFT model, by using restricted cubic splines. 

The article is organized as follows. In Section 2, we first show that an AFT model has some desirable properties, including collapsibility, that are not exhibited by a proportional hazards model. In Section 3, we derive the proposed model framework and describe the estimation process within a likelihood framework. In Section 4, we conduct a simulation study to evaluate the finite sample performance of the proposed model under complex scenarios, comparing to standard parametric AFT models. In Section 5, we illustrate the model using data from the England and Wales breast cancer registry. Finally, in Section 6, we conclude the article with a discussion. 

## 2. Causal interpretation of the aft model

**Fig. 1:**
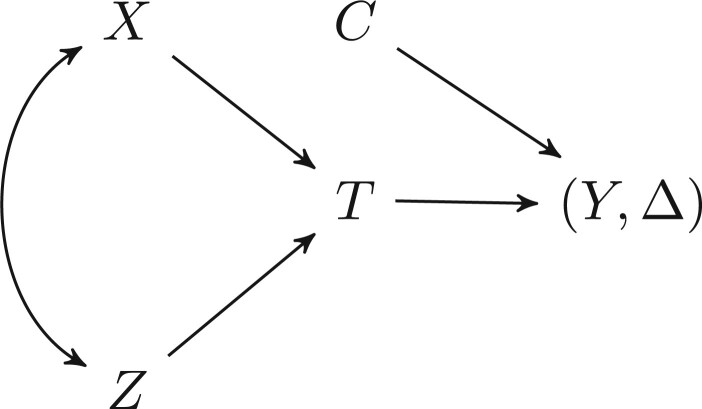
Causal diagram for the motivating example, with censoring variable $C$ being independent from $X$, $Z,$ and $T$.

Hougaard (1999) provided an informal description of how AFT models are robust to omitted covariates. We now provide a more formal development for the collapsibility of the acceleration factor for AFT models. Consider a model with two covariates $X$ and $Z$, with an event time $T$, with regression parameters $\beta_X$ and $\beta_Z$ and linear predictor $\beta_X x + \beta_Z z$. Assume that the censoring variable $C$ is independent of $X$, $Z$ and $T$, and that the time process is observed by the tuple $(Y=\min(T,C),\Delta=I(T\leq C))$; the associated causal diagram is given in Figure 1. 

Following Martinussen and Vansteelandt (2013), we define the marginal unadjusted effect for binary $X=1$ compared with $X=0$ at time $t$ for a contrast function $g$ and a prediction function $\psi$ as $\mathcal{T}_m(t)=g(\psi(t|x=1),\psi(t|x=0))$. The marginal exposure effect (causal effect) is defined as $\mathcal{T}(t)=g(\psi(t|\hat{x}=1),\psi(t|\hat{x}=0)$, where $\hat{x}=x$ is the *do* operator, which can be conceptualized as the population value that would be realized if $X$ were uniformly set to $x$. In this context, the causal effect is calculated by an expectation over $Z$ for a fixed and possibly counterfactual $X=x$, such that $\psi(t|\hat{x}=x)=E_Z(\psi(t|X=x,Z))$. For further details on the *do* operator, see Pearl (2000). 

### 2.1. Proportional hazard model

Martinussen and Vansteelandt (2013) considered a proportional hazards model $h(t|x,z)=h_0(t)\exp(\beta_X x + \beta_Z z)$. The marginal hazard conditional on survival has previously been shown to be

$$\begin{align*}
E_Z(h(t|x,Z)|T>t) &= \frac{E_Z(h(t|x,Z) S(t|x,Z))}{E_Z(S(t|x,Z))} \\
&= \frac{h_0(t)\exp(\beta_X x) E_Z(\exp(\beta_Z Z) S(t|x,Z))}{E_Z(S(t|x,Z))}
\end{align*}$$

For the marginal (causal) effect for the log-hazard ratio, we define $\psi(t|\hat{x}=x)=E_Z(h(t|\hat{x}=x,Z)|T>t)$ and $g(a,b)=\log(a/b)$, then the marginal (causal) effect is

(2.1) \begin{align*} \mathcal{T}(t) &= \log(E_Z(h(t|\hat{x}=1,Z)|T>t))-\log(E_Z(h(t|\hat{x}=0,Z)|T>t)) \nonumber \\ &= \beta_X + \log\left(\frac{E_Z(\exp(\beta_Z Z) S(t|\hat{x}=1,Z))}{E_Z(S(t|\hat{x}=1,Z))}\right) - \log\left(\frac{E_Z(\exp(\beta_Z Z) S(t|\hat{x}=0,Z))}{E_Z(S(t|\hat{x}=0,Z))}\right) \end{align*}

For a marginal unadjusted effect, we define $\psi(t|x)=E_Z(h(t|x,Z)|T>t,X=x)$. Then

(2.2) \begin{align*} \mathcal{T}_m(t) &= \log(E_Z(h(t|x=1,Z)|T>t,X=1))-\log(E_Z(h(t|x=0,Z)|T>t,X=0)) \nonumber \\ &= \beta_X + \log\left(\frac{E_Z(\exp(\beta_Z Z) S(t|x=1,Z) | X=1)}{E_Z(S(t|x=1,Z)|X=1)}\right) - \log\left(\frac{E_Z(\exp(\beta_Z Z) S(t|x=0,Z) | X=0)}{E_Z(S(t|x=0,Z) | X=0)}\right) \end{align*}

These expressions show that $\beta_X$ is a biased estimator of both the marginal causal effect and the marginal unadjusted effect. We can use (2.1) to estimate the causal effect $\hat{\mathcal{T}}(t)$ from a model fit incorporating both $X$ and $Z$, and use (2.1) and (2.2) to calculate the confounding bias $\mathcal{T}(t)-\mathcal{T}_m(t)$. We can also use (2.2) to calculate the bias in the unadjusted estimate for $\beta_X$ when $Z$ is not modeled compared with modeling both $X$ and $Z$ (that is, $\mathcal{T}_m(t)-\beta_X$) when $\beta_Z \neq 0$ and $X$ and $Z$ are associated. 

### 2.2. AFT model

Now define an AFT model $\log(T) = \alpha -(\beta_X x + \beta_Z z) + \epsilon$, where $\alpha=-\beta_0$ and $E(\epsilon)=0$. For the marginal (causal) effect for the mean time to event comparing $X=1$ with $X=0$, define $\psi(t|x)=E_Z(\log(T)|\hat{x}=x,Z)$ and $g(a,b)=a-b$, such that

$$\begin{align*}
\mathcal{T} &= E_Z(\log(T)|\hat{x}=1,Z)-E_Z(\log(T)|\hat{x}=0,Z) = -\beta_X
\end{align*}$$

which is unbiased and indicates *collapsibility* of the acceleration factor. For the marginal unadjusted effect, let $\psi(t|x)=E_Z(\log(T)|X=x,Z)$, so that

$$\begin{align*}
\mathcal{T}_m = -\beta_X - \beta_Z(E_Z(Z|X=1)-E_Z(Z|X=0))
\end{align*}$$

which will be biased if, again, $\beta_Z\neq 0$ and $X$ and $Z$ are associated. We can extend this finding to a time-dependent AFT model $S(t|x,z)=S_0\left(\int_0^t \exp(\eta(u|x,z)) {\rm d}u\right)$ for a time-varying acceleration factor $\eta(u|x,z)=-\beta_X(u) x - \beta_Z(u) z$ and for a baseline survival function $S_0(t)$. The marginal value for $\eta(t|x,Z)$ conditional on survival is

$$\begin{align*}
E_Z(\eta(t|x,Z)| T>t) &= -\beta_X(t) x - \beta_Z(t) E_Z(Z|x)
\end{align*}$$

then the marginal (causal) effect comparing $X=1$ with $X=0$ at time $t$ is

$$\begin{align*}
\mathcal{T}(t) &= E_Z(\eta(t|\hat{x}=1,Z)) - E_Z(\eta(t|\hat{x}=0,Z)) = -\beta_X(t)
\end{align*}$$

For the marginal unadjusted effect, let $\psi(t|x)=E_Z(\eta(t|X=x,Z))$, and then

$$\begin{align*}
\mathcal{T}_m(t) &= -\beta_X(t) - \beta_Z(t) (E_Z(Z|X=1) - E_Z(Z|X=0))
\end{align*}$$

Pleasantly, if $E_Z(Z|X=1) - E_Z(Z|X=0)$ is small, then $\mathcal{T}_m(t) \approx -\beta_X(t)$ and the AFT can be shown to be robust to omitted covariates. 

## 3. A general parametric aft model

Continuing with the notation defined in the previous section, an AFT model, conditional on a set of explanatory variables, $\boldsymbol{X}$, can be written in the form of the survival function,

\begin{equation*} S(t | \boldsymbol{X}) = S_{0} (t\, \phi(\boldsymbol{X};\,\boldsymbol\beta)), \nonumber \end{equation*}

where often

(3.3) \begin{equation*} \phi(\boldsymbol{X};\,\boldsymbol\beta) = \exp(-\boldsymbol{X} \boldsymbol{\beta}). \end{equation*}

We can also specify an AFT model in terms of the cumulative hazard function

(3.4) \begin{equation*} H(t | \boldsymbol{X}) = H_{0} (t\, \phi(\boldsymbol{X};\,\boldsymbol\beta)). \end{equation*}

In essence, we can specify any parametric function for (3.4), subject to the appropriate constraints that the function remains positive for all $t>0$ and is monotonically increasing as $t \rightarrow \infty$. In this article, we concentrate on a highly flexible way of specifying a parametric AFT, using restricted cubic splines as our basis functions (Durrleman and Simon, 1989). 

Similarly to Royston and Parmar (2002), we begin with the log cumulative hazard function of the Weibull distribution,

\begin{equation*} \log H(t|\lambda,\zeta) = \log (\lambda) + \zeta \log (t), \quad \lambda, \zeta > 0 \nonumber \end{equation*}

Instead of incorporating covariates into the linear predictor of the $\log(\lambda)$ component, as in Royston and Parmar (2002), here, we incorporate them as a multiplicative effect on $t$,

\begin{equation*} \log H(t | \boldsymbol{X},\lambda, \zeta,\boldsymbol\beta) = \log (\lambda) + \zeta \log (t\, \phi(\boldsymbol{X};\,\boldsymbol\beta)), \nonumber \end{equation*}

where $\phi(\boldsymbol{X};\,\boldsymbol\beta)$ is defined in (3.3). Now we can incorporate the desired flexibility, expanding $\log (t\, \phi(\boldsymbol{X};\,\boldsymbol\beta))$ into restricted cubic spline basis. For simplicity, letting $u = \log (t\, \phi(\boldsymbol{X};\,\boldsymbol\beta))$, our spline function is defined as

\begin{equation*} s(u|\boldsymbol{\gamma},\boldsymbol{k_{0}}) = \gamma_{0} + \gamma_{1}v_{1}(u,\boldsymbol{k_{0}}) + \gamma_{2}v_{2}(u,\boldsymbol{k_{0}}) + \dots + \gamma_{m+1}v_{m+1}(u,\boldsymbol{k_{0}}), \nonumber \end{equation*}

where $\boldsymbol{k}_{0}$ is a vector of knot locations with parameter vector $\boldsymbol{\gamma}$, and derived variables $v_{j}$ (known as the basis functions). For a truncated power basis, the $v_j$ are defined as

\begin{align*} v_{1}(u,\boldsymbol{k_{0}}) &= u \nonumber\\ v_{j}(u,\boldsymbol{k_{0}}) &= (u-k_{j})^{3}_{+} - \lambda_{j}(u-k_{\text{min}})^{3}_{+} - (1-\lambda_{j})(u-k_{\text{max}})^{3}_{+}, \nonumber \end{align*}

where for $j=2,\dots,m+1$, $(u-k_{j})^{3}_{+}$ is equal to $(u-k_{j})^{3}$ if the value is positive and 0 otherwise, and

\begin{equation*} \lambda_{j} = \frac{k_{\text{max}}-k_{j}}{k_{\text{max}}-k_{\text{min}}}. \nonumber \end{equation*}

Alternatively, the $v_j(u,\boldsymbol{k_{0}})$ can be calculated using a B-spline basis with a matrix projection at the boundary knots. Given one of these bases, our flexible parametric AFT model can be defined as

$$\log H(t | \boldsymbol{X}) = s(\log(t\, \phi(\boldsymbol{X};\,\boldsymbol\beta)) | \boldsymbol{\gamma},\boldsymbol{k}_{0}).
$$

Usually, knot locations are calculated based on quantiles of the distribution of the variable being transformed into splines, in this case $\log(t\, \phi(\boldsymbol{X};\,\boldsymbol\beta))$, also restricted to those observations which are uncensored. 

### 3.1. Likelihood and estimation

We define the likelihood in terms of the hazard and survival functions. The hazard function can be written as follows

\begin{align*} h(t|\boldsymbol X) &= \frac{{\rm d}}{{\rm d}t} H(t|\boldsymbol X) \nonumber \\ &= \exp[s(\log(t\,\phi(\boldsymbol{X};\,\boldsymbol\beta))|\boldsymbol{\gamma},\boldsymbol{k}_{0})] \times \frac{{\rm d}}{{\rm d} t} [ s(\log(t\,\phi(\boldsymbol{X};\,\boldsymbol\beta))|\boldsymbol{\gamma},\boldsymbol{k}_{0}) ] \nonumber \\ &= \exp[s(\log(t\,\phi(\boldsymbol{X};\,\boldsymbol\beta))|\boldsymbol{\gamma},\boldsymbol{k}_{0})] \times \frac{1}{t} \times s^{\prime}(\log(t\,\phi(\boldsymbol{X};\,\boldsymbol\beta))|\boldsymbol{\gamma},\boldsymbol{k}_{0}), \nonumber \end{align*}

where $s^{\prime}(x) = \frac{{\rm d}}{{\rm d}x}s(x)$, which can be readily derived analytically. The survival function is defined as

\begin{equation*} S(t|\boldsymbol X) = \exp[-\exp\{s(\log(t\,\phi(\boldsymbol{X};\,\boldsymbol\beta))|\boldsymbol{\gamma},\boldsymbol{k}_{0})\}]. \nonumber \end{equation*}

We can therefore define our log likelihood for the $i{\rm th}$ patient, allowing for delayed entry, as

(3.5) \begin{align*} l_{i} &= \delta_{i} \log h(y_{i}) + \log S(y_{i}) - \log S(t_{0i}) \nonumber \\ &= \delta_{i} \times \left[ s(\log(y_{i}\,\phi(\boldsymbol{X};\,\boldsymbol\beta))|\boldsymbol{\gamma},\boldsymbol{k}_{0}) -\log(y_{i}) + \log(s^{\prime}(\log(y_{i}\,\phi(\boldsymbol{X};\,\boldsymbol\beta))|\boldsymbol{\gamma},\boldsymbol{k}_{0})) \right] \nonumber \\ &\qquad \quad -\exp \{s(\log(y_{i}\,\phi(\boldsymbol{X};\,\boldsymbol\beta))|\boldsymbol{\gamma},\boldsymbol{k}_{0}) \} + \exp \{s(\log(t_{0i}\,\phi(\boldsymbol{X};\,\boldsymbol\beta))|\boldsymbol{\gamma},\boldsymbol{k}_{0})\}, \end{align*}

where $\delta_i$ is an event indicator with value 1 for an event and 0 for censored. We maximize (3.5) using Newton–Raphson based optimization (Gould *and others*, 2010), with analytic score and Hessian for both accuracy and efficiency.

### 3.2. Time-dependent acceleration factors

Following Cox and Oakes (1984) and Hougaard (1999), we have the survival function of a time-dependent AFT model, such that

$$S(t) = S_{0} \left( \int_{0}^{t} \eta(\boldsymbol X, u;\,\boldsymbol\beta) \text{ d} u \right)\!,
$$

where $\eta(\boldsymbol X, u;\,\boldsymbol\beta)$ is the time-varying acceleration factor at time $u$ and the baseline survival is $S_0(t)=\exp(-\exp(s(\log(t)|\boldsymbol{\gamma},\boldsymbol{k}_{0})))$. To simplify the notation in this section, we have dropped the dependence on individual $i$. Within our flexible parametric framework, we can avoid the integration by directly modeling on the cumulative scale, such that

$$S_{0} \left( \int_{0}^{t} \eta(\boldsymbol X, u;\,\boldsymbol\beta) \text{ d} u \right) = S_{0}(t \times \phi(\boldsymbol X, t;\,\boldsymbol\beta)).
$$

Since we are on a cumulative scale, to recover the directly interpretable time-dependent acceleration factor, $\eta(\boldsymbol X, t;\,\boldsymbol\beta)$, we derive the following relationship,

(3.6) \begin{align*} \int_{0}^{t} \eta(\boldsymbol X, u;\,\boldsymbol\beta) \text{ d} u &= t \times \phi(\boldsymbol{X}, t;\,\boldsymbol\beta) \nonumber \\ \implies \eta(\boldsymbol X, t;\,\boldsymbol\beta) &= \frac{{\rm d}}{{\rm d} t} \left[ t \phi(\boldsymbol{X},t;\,\boldsymbol\beta) \right] \nonumber \\ &= \phi(\boldsymbol{X},t;\,\boldsymbol\beta) + t\,\frac{{\rm d}}{{\rm d} t} \phi(\boldsymbol{X}, t;\,\boldsymbol\beta) \end{align*}

which gives a rather convenient formula for the time-dependent acceleration factor in terms of its cumulative. We can arguably use any continuous function to capture simple and complex time-dependent acceleration factors. The hazard function is defined as

(3.7) \begin{align*} h(t| \boldsymbol X) &= \exp[s(\log(t \phi(\boldsymbol{X},t))|\boldsymbol{\gamma},\boldsymbol{k}_{0})] \times \frac{\rm d }{{\rm d} t} [ s(\log(t \phi(\boldsymbol{X},t))|\boldsymbol{\gamma},\boldsymbol{k}_{0}) ] \nonumber \\ &= \exp[s(\log(t \phi(\boldsymbol{X},t))|\boldsymbol{\gamma},\boldsymbol{k}_{0})] \times \frac{t \frac{{\rm d} \phi(\boldsymbol{X},t)}{{\rm d} t} + \phi(\boldsymbol{X},t) }{t \phi(\boldsymbol{X},t)} \times s^{\prime}(\log(t \phi(\boldsymbol{X},t))|\boldsymbol{\gamma},\boldsymbol{k}_{0}). \end{align*}

Our form of choice continues the use of restricted cubic splines. A common case is to use a minus log link for the linear predictor, such that

(3.8) \begin{equation*} \phi(\boldsymbol{X},t;\,\boldsymbol\beta) = \exp\left( -\boldsymbol{X} \boldsymbol{\beta} - \sum_{p=1}^{P} x_{p} s(\log(t)| \boldsymbol{\gamma}_{p}, \boldsymbol{k}_{p})\right)\!, \end{equation*}

where for the $p{\rm th}$ time-dependent effect, with $p = \{1,\dots,P\}$, we have $x_{p}$, the $p{\rm th}$ covariate, multiplied by some spline function of log time, $s(\log(t)| \boldsymbol{\gamma}_{p},\boldsymbol{k}_{p})$, with knot location vector, $\boldsymbol{k}_{p}$, and coefficient vector, $\boldsymbol{\gamma}_{p}$.

Now

(3.9) \begin{align*} \frac{{\rm d} \phi(\boldsymbol{X},t;\,\boldsymbol\beta)}{{\rm d} t} &= \frac{{\rm d}}{{\rm d} t} \exp\left( -\boldsymbol{X} \boldsymbol{\beta} - \sum_{p=1}^{P} x_{p} s(\log(t)| \boldsymbol{\gamma}_{p}, \boldsymbol{k}_{p})\right) \nonumber \\ &= \phi(\boldsymbol{X},t;\,\boldsymbol\beta) \times \frac{{\rm d}}{{\rm d} t} \left[ -\boldsymbol{X} \boldsymbol{\beta} - \sum_{p=1}^{P} x_{p} s(\log(t)| \boldsymbol{\gamma}_{p}, \boldsymbol{k}_{p}) \right] \nonumber \\ &= \phi(\boldsymbol{X},t;\,\boldsymbol\beta) \times \left[ -\sum_{p=1}^{P} x_{p} \frac{{\rm d} \log(t)}{{\rm d} t} \frac{{\rm d}}{{\rm d} \log(t)} s(\log(t)| \boldsymbol{\gamma}_{p}, \boldsymbol{k}_{p}) \right] \nonumber \\ &= \frac{\phi(\boldsymbol{X},t;\,\boldsymbol\beta)}{t} \times \left[ -\sum_{p=1}^{P} x_{p} s^{\prime}(\log(t)| \boldsymbol{\gamma}_{p}, \boldsymbol{k}_{p}) \right]. \end{align*}

Substituting (3.9) into (3.7)

(3.10) \begin{align*} h(t|\boldsymbol X) &= \exp[s(\log(t \phi(\boldsymbol{X},t;\,\boldsymbol\beta))|\boldsymbol{\gamma},\boldsymbol{k}_{0})] \times s^{\prime}(\log(\phi(\boldsymbol{X},t;\,\boldsymbol\beta))|\boldsymbol{\gamma},\boldsymbol{k}_{0}) \times \frac{1}{t} \times \left[\!1 - \sum_{p=1}^{P} x_{p} s^{\prime}(\log(t)| \boldsymbol{\gamma}_{p}, \boldsymbol{k}_{p})\!\right] \end{align*}

with survival function

(3.11) \begin{equation*} S(t|\boldsymbol X) = \exp[-\exp\{s(\log(t\phi(\boldsymbol{X},t;\,\boldsymbol\beta))|\boldsymbol{\gamma},\boldsymbol{k}_{0})\}]. \end{equation*}

Equations (3.10) and (3.11) can then be substituted into (3.5) to maximize the log likelihood. Analytic scores and Hessian elements are also derived and implemented in the associated software packages. 

## 4 Simulations

### 4.1. Causal inference

We first simulate under Figure 1. Assume that $X$ is Bernoulli, $Z$ is normal, $T$ is exponential and $C$ is uniform. Specifically, let $X\sim\text{Bernoulli}(0.5), Z\sim\text{Normal}(0,2^2), T\sim\text{Exponential}(\exp(\beta_0+\beta_XX+\beta_ZZ)), \beta_0=-5, \beta_X=\beta_Z=1, C\sim\text{Uniform}(0,10)$ and $(Y,\Delta)=(\min(T,C),T<C)$. Let $(x,z,y,\delta)$ be realizations for $(X,Z,Y,\Delta)$. These data can be modeled using both proportional hazards and AFT models. We fit models for $(y,\delta)$ with both $x$ and $z$ as linear and additive covariates and with only $x$ as a covariate. We model using Poisson regression, Cox regression and our smooth AFT with 3 degrees of freedom (see Table 1). Note that the estimated $\beta_X$’s have opposite signs for the AFT models compared with the proportional hazards models (Poisson and Cox regression). As a reminder, an exponential AFT would estimate $-\beta_X$ as per the Poisson regression. We find that all of the models are unbiased when both covariates are included ($|E(\hat\beta_X)|=1$). 

**Table 1:** Simulation results for exponentially distributed data with $\beta_X=1$, with $n=10^4$ observations per simulation and 300 simulation sets

		$(y,\delta) \sim x+z$			$(y,\delta)\sim x$	
$\text{Corr}(X,Z)$	Model	$E(\hat\beta_X)$	$E(se(\hat\beta_X))$		$E(\hat\beta_X)$	$E(se(\hat\beta_X))$
0	Poisson regression	0.998	0.054		0.679	0.053
	Cox regression	0.999	0.054		0.663	0.053
	Smooth AFT	$-$ 0.998	0.055		$-$ 0.962	0.079
0.1	Poisson regression	0.998	0.054		0.901	0.054
	Cox regression	0.999	0.055		0.880	0.054
	Smooth AFT	$-$ 0.998	0.056		$-$ 1.278	0.083
$-$ 0.1	Poisson regression	0.998	0.053		0.464	0.052
	Cox regression	0.999	0.054		0.454	0.052
	Smooth AFT	$-$ 0.998	0.055		$-$ 0.657	0.077

The regression models assume that both covariates are modeled ($(y,\delta) \sim x+z$) or that only the $x$ covariate is modeled ($(y,\delta)\sim x$). The expectations for the estimated $\beta_X$ and their standard errors are over the simulation sets.

When $X$ and $Z$ are independent, then the effect of $X$ is not confounded by $Z$ and $\mathcal{T}_m(t)=\mathcal{T}(t)$. For the AFT models, assuming that we have captured the baseline distribution, then $E_Z(Z|X=1)=E_Z(Z|X=0)$ and $\mathcal{T}_m(t)=-\beta_X(t)$. However, for the proportional hazards models with both covariates, the marginal (causal) effect is attenuated for increasing time. This pattern of attenuation is well recognized from the context of frailty models (e.g., Aalen *and others*, 2008). Moreover, when the covariate z is not included and X and Z are negatively correlated, then the first two models are more attenuated. When X and Z are positively correlated and z was not included, then the first two models had attenuated effects for $x$, while the AFT model had an inflated effect and a larger bias. 

### 4.2. Other simulations

In this section, we conduct a simulation study to assess the ability of the flexible parametric AFT model to capture complex, biologically plausible, baseline functions, and subsequently the impact on estimates of acceleration factors and survival probabilities, when misspecifying the baseline. We also compare the newly proposed flexible AFT to existing parametric models, including the Weibull, generalized gamma, and generalized F. The Weibull and generalized gamma models are available in the streg command in Stata, and we implement the generalized F in Stata, following Cox (2008). 

In all simulations, we use a range of two-component mixture Weibull baseline hazard functions, and also a standard Weibull, to generate complex, realistic scenarios (Crowther and Lambert, 2013). When fitting the flexible parametric models, we are therefore not fitting the “true” model, but investigating how well the spline approximations can do (Rutherford *and others*, 2015). The baseline survival function for a two-component mixture Weibull is defined as follows:

(4.12) \begin{equation*} S_{0}(t) = p \exp (-\lambda_{1}t^{\gamma_{1}}) + (1-p) \exp(-\lambda_{2}t^{\gamma_{2}}). \end{equation*}

**Fig. 2:**
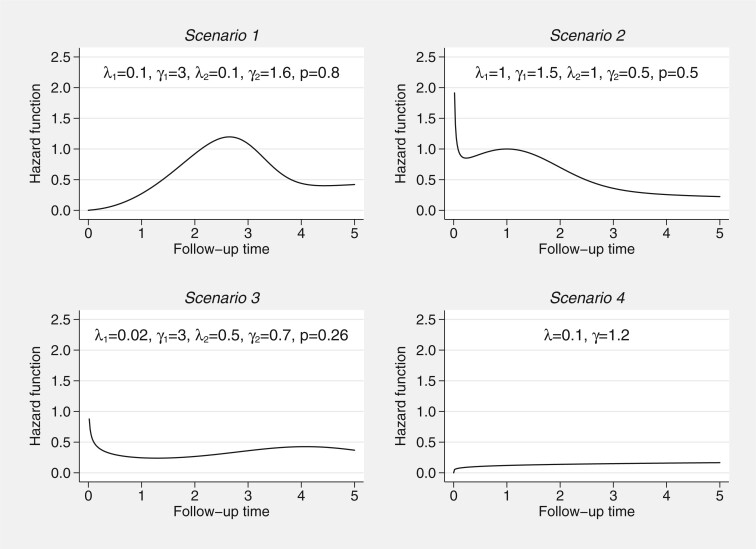
Baseline hazard functions for the simulation scenarios.

We choose four different baseline hazard functions, representing clinically plausible functions (Royston and Lambert, 2011; Murtaugh *and others*, 1994). The four assumed baseline hazard functions are shown in Figure 2. Scenarios 1–3 come from mixture Weibull functions defined in (4.12), with Scenario 4 a standard Weibull function. With our baseline functions defined, we can choose to simulate under an AFT framework, or under proportional hazards, using the general survival simulation framework developed by Crowther and Lambert (2013). 

Consider a binary treatment group variable, $X$, with a log acceleration factor, $\beta$. We can simulate AFT data from the following,

(4.13) \begin{align*} S(t| X) &= p \exp (-\lambda_{1}(t e^{-X\beta})^{\gamma_{1}}) + (1-p) \exp(-\lambda_{2}(t e^{-X\beta})^{\gamma_{2}}) \nonumber \\ &= p \exp (-\lambda_{1}t^{\gamma_{1}} e^{-X\beta\gamma_{1}}) + (1-p) \exp(-\lambda_{2}t^{\gamma_{2}} e^{-X\beta\gamma_{2}}). \end{align*}

For each scenario, we assume a log AF of $\beta=-0.5$, or $\beta=0.5$. This results in 8 scenarios in total. 

To each simulated data set, we apply a Weibull AFT model, a generalized gamma AFT model, a generalized F AFT model and the proposed flexible parametric AFT model with 2 to 9 degrees of freedom. We do not fit a flexible parametric acceleration failure time model with 1 degree of freedom, as this is equivalent to a Weibull AFT model. Each simulation scenario is repeated 1000 times with 1000 observations in each data set. We set a maximum follow-up time of 5 years. The following average survival probability at 5 years was observed in each scenario; $\bar{S}(5)=0.03$ in scenario 1 and $\beta=-0.5$, $\bar{S}(5)=0.106$ in Scenario 1 and $\beta=0.5$, $\bar{S}(5)=0.040$ in Scenario 2 and $\beta=-0.5$, $\bar{S}(5)=0.071$ in scenario 1 and $\beta=0.5$, $\bar{S}(5)=0.131$ in Scenario 3 and $\beta=-0.5$, $\bar{S}(5)=0.289$ in Scenario 3 and $\beta=0.5$, $\bar{S}(5)=0.393$ in Scenario 4 and $\beta=-0.5$, $\bar{S}(5)=0.592$ in Scenario 4 and $\beta=0.5$. 

We monitor estimates of $\beta$ from all models, and estimates of the survival probability at 1, 2, 3, 4, and 5 years, in both treatment groups. Survival was monitored on the $\log [-\log()]$ scale, with standard errors calculated using the delta method. We also monitor values of the Akaike Information Criterion (AIC) and Bayesian Information Criterion (BIC). 

### 4.3. Simulation results

Results are presented in Table 2 for all eight scenarios. We present bias, percentage bias, and coverage for the estimates of the log acceleration factor from all AFT models. We further present the median rank in terms of best fitting model based on either the AIC or BIC, for all models fitted. Finally, in Tables 3–6 we present bias, percentage bias, and coverage for estimates of the survival probability at 1, 2, 3, 4, and 5 years, for the four scenarios, when $X=0$ and $\beta=0.5$, $X=1$ and $\beta=0.5$, $X=0$ and $\beta=-0.5$, $X=1$ and $\beta=-0.5$, respectively, with all estimates are on the $\log \left\{-\log[S(t)]\right\}$ scale. 

**Table 2:** Simulation results

True log(AF)	Model	Scenario 1						Scenario 2						Scenario 3						Scenario 4					
		Bias	$\%$ Bias	Cov.	AIC	BIC	$\#$ Conv.	Bias	$\%$ Bias	Cov.	AIC	BIC	$\#$ Conv.	Bias	$\%$ Bias	Cov.	AIC	BIC	$\#$ Conv.	Bias	$\%$ Bias	Cov.	AIC	BIC	$\#$ Conv.
0.5	Weibull	$-$ 0.083	$-$ 16.6	23.4	11	11	1000	$-$ 0.049	$-$ 9.8	90.6	11	10	1000	0.139	27.8	67.6	11	10	1000	0.002	0.4	95.4	1	1	1000
	Gamma	$-$ 0.033	$-$ 6.6	81.6	10	10	1000	$-$ 0.074	$-$ 14.8	83.8	10	10	1000	0.034	6.8	65.0	6	2	684	0.003	0.6	87.5	3	3	915
	GenF	0.000	0.0	94.5	8	3	996	0.001	0.2	66.9	7	1	701	0.036	7.2	33.8	5	3	360	0.013	2.6	55.5	5	5	587
	FPAFT-df=2	$-$ 0.001	$-$ 0.2	94.1	9	9	1000	$-$ 0.077	$-$ 15.4	81.0	9	9	1000	0.102	20.4	76.3	10	7	1000	0.004	0.8	95.4	3	3	1000
	FPAFT-df=3	0.001	0.2	94.3	1	1	1000	0.047	9.4	91.1	8	7	1000	0.002	0.4	93.9	2	2	1000	0.003	0.6	95.3	5	4	1000
	FPAFT-df=4	0.000	0.0	95.4	2	2	1000	$-$ 0.001	$-$ 0.2	93.2	4	1	1000	0.002	0.4	92.5	3	4	1000	0.002	0.4	94.7	6	6	1000
	FPAFT-df=5	0.002	0.4	95.1	3	4	1000	$-$ 0.002	$-$ 0.4	93.9	3	3	1000	0.008	1.6	94.1	4	5	1000	0.000	0.0	92.8	7	7	1000
	FPAFT-df=6	0.001	0.2	94.0	4	5	1000	$-$ 0.004	$-$ 0.8	93.8	3	4	1000	0.007	1.4	93.5	5	6	1000	0.001	0.2	88.2	8	8	1000
	FPAFT-df=7	0.000	0.0	93.8	5	6	1000	$-$ 0.002	$-$ 0.4	92.5	3	5	1000	0.003	0.6	93.6	6	8	1000	$-$ 0.001	$-$ 0.2	85.1	9	9	1000
	FPAFT-df=8	$-$ 0.000	0.0	92.4	6	7	999	0.000	0.0	89.6	4	6	1000	0.002	0.4	90.2	7	9	1000	$-$ 0.004	$-$ 0.8	78.1	10	10	1000
	FPAFT-df=9	$-$ 0.000	0.0	89.9	7	8	998	$-$ 0.001	$-$ 0.2	88.8	5	8	1000	0.003	0.6	88.5	8	11	1000	$-$ 0.009	$-$ 1.8	72.0	11	11	1000
$-$ 0.5	Weibull	0.057	$-$ 11.4	57.6	11	11	1000	0.042	$-$ 8.4	91.1	10	10	1000	0.009	$-$ 1.8	96.5	10	10	1000	$-$ 0.000	0.0	95.9	1	1	1000
	Gamma	0.010	$-$ 2.0	93.1	9	9	989	0.047	$-$ 9.4	88.8	10	11	1000	0.079	$-$ 15.8	76.4	8	4	1000	$-$ 0.001	0.2	95.6	3	3	998
	GenF	0.000	0.0	94.4	6	1	993	0.000	0.0	64.8	6	1	679	$-$ 0.003	0.6	38.2	1	1	397	$-$ 0.007	1.4	69.9	5	5	732
	FPAFT-df=2	0.053	$-$ 10.6	62.5	10	10	1000	0.039	$-$ 7.8	88.5	9	9	1000	0.040	$-$ 8.0	92.0	9	6	1000	$-$ 0.001	0.2	95.7	3	3	1000
	FPAFT-df=3	0.040	$-$ 8.0	67.1	8	7	1000	0.112	$-$ 22.4	72.4	8	8	1000	0.041	$-$ 8.2	90.1	5	1	1000	$-$ 0.001	0.2	96.3	5	4	1000
	FPAFT-df=4	0.022	$-$ 4.4	86.7	6	3	1000	0.061	$-$ 12.2	82.6	6	2	1000	0.048	$-$ 9.6	87.4	6	3	1000	$-$ 0.001	0.2	95.6	6	6	1000
	FPAFT-df=5	0.012	$-$ 2.4	91.5	4	2	1000	0.038	$-$ 7.6	89.6	5	2	1000	0.058	$-$ 11.6	81.3	5	4	1000	$-$ 0.002	0.4	93.6	7	7	1000
	FPAFT-df=6	0.005	$-$ 1.0	93.2	2	4	1000	0.023	$-$ 4.6	92.2	3	3	1000	0.062	$-$ 12.4	78.6	4	6	1000	$-$ 0.002	0.4	90.2	8	8	1000
	FPAFT-df=7	0.001	$-$ 0.2	93.1	3	5	1000	0.011	$-$ 2.2	91.9	2	5	1000	0.061	$-$ 12.2	74.8	3	7	1000	$-$ 0.002	0.4	86.8	9	9	1000
	FPAFT-df=8	$-$ 0.003	0.6	92.3	3	6	1000	0.004	$-$ 0.8	89.6	3	6	1000	0.059	$-$ 11.8	73.4	3	8	1000	0.002	$-$ 0.4	80.1	10	10	1000
	FPAFT-df=9	$-$ 0.004	0.8	90.4	4	8	1000	0.001	$-$ 0.2	89.5	4	7	1000	0.057	$-$ 11.4	71.9	3	9	1000	0.007	$-$ 1.4	75.0	11	11	1000

**Table 3:** Bias, percentage bias, and coverage of estimates of log($-$log(S(t))) when $X=0$ and $\beta=0.5$

Time	Model	Scenario 1				Scenario 2				Scenario 3				Scenario 4			
		Bias	$\%$ Bias	Cov.	$\#$ Conv.	Bias	$\%$ Bias	Cov.	$\#$ Conv.	Bias	$\%$ Bias	Cov.	$\#$ Conv.	Bias	$\%$ Bias	Cov.	$\#$ Conv.
1	Weibull	0.218	$-$ 9.5	19.6	1000	$-$ 0.020	.	92.2	1000	0.136	$-$ 13.0	36.2	1000	$-$ 0.009	0.4	95.2	1000
2	Weibull	$-$ 0.154	39.8	15.7	1000	$-$ 0.134	$-$ 21.1	18.4	1000	0.185	$-$ 36.0	4.5	1000	$-$ 0.006	0.4	95.8	1000
3	Weibull	$-$ 0.224	$-$ 38.2	1.4	1000	$-$ 0.065	$-$ 7.4	70.7	1000	0.106	$-$ 110.5	40.2	1000	$-$ 0.004	0.4	96.2	1000
4	Weibull	0.082	8.9	57.8	1000	0.035	3.5	86.7	1000	$-$ 0.019	$-$ 7.1	95.7	1000	$-$ 0.003	0.5	96.4	1000
5	Weibull	0.427	39.8	0.0	1000	0.118	11.0	39.1	1000	$-$ 0.104	$-$ 19.2	47.6	1000	$-$ 0.002	0.5	96.1	1000
1	Gamma	0.063	$-$ 2.7	87.3	1000	$-$ 0.056	.	79.9	1000	0.007	$-$ 0.7	87.7	929	$-$ 0.006	0.3	95.3	1000
2	Gamma	$-$ 0.027	7.0	94.0	1000	$-$ 0.146	$-$ 22.9	14.6	1000	0.048	$-$ 9.3	77.2	929	$-$ 0.007	0.5	94.4	1000
3	Gamma	$-$ 0.143	$-$ 24.4	14.4	1000	$-$ 0.051	$-$ 5.8	77.7	1000	0.018	$-$ 18.8	86.0	929	$-$ 0.007	0.7	93.9	1000
4	Gamma	0.056	6.1	72.4	1000	0.074	7.5	68.1	1000	$-$ 0.024	$-$ 8.9	83.2	929	$-$ 0.006	0.9	95.2	1000
5	Gamma	0.283	26.4	0.1	1000	0.180	16.7	16.1	1000	0.005	0.9	88.4	929	$-$ 0.002	0.5	96.1	1000
1	GenF	0.006	$-$ 0.3	92.9	990	0.001	.	67.4	707	0.007	$-$ 0.7	25.7	295	$-$ 0.006	0.3	69.4	779
2	GenF	0.004	$-$ 1.0	95.6	997	$-$ 0.018	$-$ 2.8	66.7	713	0.049	$-$ 9.5	31.3	380	0.000	0.0	71.0	801
3	GenF	$-$ 0.037	$-$ 6.3	85.1	997	$-$ 0.003	$-$ 0.3	67.4	713	0.042	$-$ 43.8	52.1	628	0.005	$-$ 0.5	73.9	841
4	GenF	0.026	2.8	88.3	997	0.028	2.8	63.9	713	0.056	20.8	54.6	868	0.011	$-$ 1.7	75.2	873
5	GenF	0.103	9.6	48.7	997	0.043	4.0	60.5	713	0.055	10.2	55.9	907	0.000	0.0	76.2	874
1	FPAFT-df=2	0.032	$-$ 1.4	87.3	1000	$-$ 0.073	.	67.5	1000	0.041	$-$ 3.9	88.6	1000	$-$ 0.009	0.4	94.5	1000
2	FPAFT-df=2	0.050	$-$ 12.9	85.6	1000	$-$ 0.140	$-$ 22.0	17.4	1000	0.128	$-$ 24.9	29.7	1000	$-$ 0.003	0.2	95.7	1000
3	FPAFT-df=2	$-$ 0.104	$-$ 17.7	37.0	1000	$-$ 0.036	$-$ 4.1	83.1	1000	0.091	$-$ 94.9	52.7	1000	$-$ 0.002	0.2	96.4	1000
4	FPAFT-df=2	0.028	3.0	85.1	1000	0.091	9.2	57.1	1000	0.001	0.4	96.3	1000	$-$ 0.002	0.3	96.7	1000
5	FPAFT-df=2	0.209	19.5	12.7	1000	0.195	18.1	9.2	1000	$-$ 0.055	$-$ 10.2	83.3	1000	$-$ 0.002	0.5	96.0	1000
1	FPAFT-df=3	0.013	$-$ 0.6	95.0	1000	0.049	.	85.5	1000	$-$ 0.001	0.1	96.6	1000	$-$ 0.007	0.3	94.6	1000
2	FPAFT-df=3	$-$ 0.005	1.3	94.1	1000	$-$ 0.058	$-$ 9.1	78.9	1000	0.002	$-$ 0.4	94.1	1000	$-$ 0.003	0.2	95.5	1000
3	FPAFT-df=3	$-$ 0.000	0.0	95.0	1000	$-$ 0.026	$-$ 3.0	90.1	1000	$-$ 0.001	1.0	93.3	1000	$-$ 0.003	0.3	96.1	1000
4	FPAFT-df=3	0.011	1.2	93.9	1000	0.037	3.7	86.6	1000	$-$ 0.012	$-$ 4.5	92.9	1000	$-$ 0.003	0.5	95.8	1000
5	FPAFTdf=3	0.007	0.7	94.3	1000	0.090	8.4	65.5	1000	0.007	1.3	94.1	1000	$-$ 0.002	0.5	95.9	1000
1	FPAFT-df=4	0.002	$-$ 0.1	95.7	1000	$-$ 0.006	.	91.8	1000	$-$ 0.010	1.0	95.1	1000	$-$ 0.006	0.3	95.0	1000
2	FPAFT-df=4	0.001	$-$ 0.3	95.7	1000	$-$ 0.003	$-$ 0.5	93.8	1000	0.008	$-$ 1.6	95.4	1000	$-$ 0.003	0.2	95.6	1000
3	FPAFT-df=4	$-$ 0.005	$-$ 0.9	94.6	1000	$-$ 0.006	$-$ 0.7	95.0	1000	$-$ 0.001	1.0	95.6	1000	$-$ 0.004	0.4	96.1	1000
4	FPAFT-df=4	0.020	2.2	91.6	1000	0.004	0.4	94.2	1000	$-$ 0.015	$-$ 5.6	93.6	1000	$-$ 0.003	0.5	95.4	1000
5	FPAFT-df=4	0.010	0.9	94.4	1000	0.008	0.7	94.3	1000	0.008	1.5	94.4	1000	$-$ 0.003	0.8	96.1	1000
1	FPAFT-df=5	0.004	$-$ 0.2	95.4	1000	$-$ 0.002	.	94.7	1000	0.001	$-$ 0.1	95.6	1000	$-$ 0.007	0.3	94.9	1000
2	FPAFT-df=5	$-$ 0.001	0.3	95.5	1000	$-$ 0.001	$-$ 0.2	93.9	1000	0.000	0.0	95.0	1000	$-$ 0.005	0.3	95.0	1000
3	FPAFT-df=5	$-$ 0.003	$-$ 0.5	95.5	1000	$-$ 0.002	$-$ 0.2	94.4	1000	0.007	$-$ 7.3	94.8	1000	$-$ 0.005	0.5	94.5	1000
4	FPAFT-df=5	0.017	1.8	93.2	1000	0.004	0.4	94.4	1000	$-$ 0.006	$-$ 2.2	94.2	1000	$-$ 0.005	0.8	94.0	1000
5	FPAFT-df=5	0.009	0.8	94.6	1000	0.005	0.5	94.1	1000	0.004	0.7	94.9	1000	$-$ 0.004	1.1	95.2	1000
1	FPAFT-df=6	$-$ 0.005	0.2	94.6	1000	$-$ 0.001	.	95.0	1000	$-$ 0.001	0.1	96.0	1000	$-$ 0.007	0.3	94.7	1000
2	FPAFT-df=6	0.000	0.0	95.1	1000	$-$ 0.002	$-$ 0.3	94.4	1000	0.001	$-$ 0.2	94.7	1000	$-$ 0.005	0.3	93.9	1000
3	FPAFT-df=6	0.002	0.3	95.1	1000	0.001	0.1	95.1	1000	0.004	$-$ 4.2	94.6	1000	$-$ 0.005	0.5	93.4	1000
4	FPAFT-df=6	0.007	0.8	93.6	1000	0.005	0.5	94.6	1000	$-$ 0.001	$-$ 0.4	94.2	1000	$-$ 0.005	0.8	93.6	1000
5	FPAFT-df=6	0.005	0.5	94.1	1000	0.001	0.1	94.0	1000	0.003	0.6	94.8	1000	$-$ 0.004	1.1	95.4	1000
1	FPAFT-df=7	$-$ 0.008	0.3	93.9	1000	$-$ 0.002	.	93.6	1000	$-$ 0.003	0.3	95.6	1000	$-$ 0.007	0.3	93.5	1000
2	FPAFT-df=7	$-$ 0.001	0.3	95.4	1000	$-$ 0.002	$-$ 0.3	93.7	1000	$-$ 0.000	0.0	94.6	1000	$-$ 0.006	0.4	92.1	1000
3	FPAFT-df=7	0.003	0.5	95.1	1000	0.002	0.2	94.2	1000	0.000	0.0	93.8	1000	$-$ 0.006	0.6	92.9	1000
4	FPAFT-df=7	0.002	0.2	93.4	1000	0.006	0.6	94.8	1000	0.001	0.4	94.0	1000	$-$ 0.006	0.9	93.1	1000
5	FPAFT-df=7	0.003	0.3	94.3	1000	0.001	0.1	94.3	1000	0.002	0.4	94.6	1000	$-$ 0.005	1.3	93.9	1000
1	FPAFT-df=8	$-$ 0.010	0.4	93.9	1000	$-$ 0.001	.	94.3	1000	$-$ 0.002	0.2	95.7	1000	$-$ 0.009	0.4	92.9	1000
2	FPAFT-df=8	$-$ 0.002	0.5	94.4	1000	$-$ 0.002	$-$ 0.3	93.0	1000	$-$ 0.000	0.0	94.2	1000	$-$ 0.007	0.5	91.0	1000
3	FPAFT-df=8	0.002	0.3	94.6	1000	0.002	0.2	94.2	1000	$-$ 0.001	1.0	93.6	1000	$-$ 0.008	0.8	91.1	1000
4	FPAFT-df=8	0.001	0.1	92.6	1000	0.006	0.6	94.8	1000	0.003	1.1	93.8	1000	$-$ 0.008	1.3	91.3	1000
5	FPAFT-df=8	0.004	0.4	94.1	1000	0.002	0.2	93.9	1000	0.002	0.4	94.4	1000	$-$ 0.006	1.6	93.8	1000
1	FPAFT-df=9	$-$ 0.011	0.5	94.3	1000	$-$ 0.001	.	92.3	1000	$-$ 0.003	0.3	95.6	1000	$-$ 0.012	0.5	91.7	1000
2	FPAFT-df=9	$-$ 0.002	0.5	93.9	1000	$-$ 0.001	$-$ 0.2	93.5	1000	$-$ 0.001	0.2	94.2	1000	$-$ 0.010	0.7	89.8	1000
3	FPAFT-df=9	0.001	0.2	94.1	1000	0.002	0.2	94.2	1000	$-$ 0.001	1.0	93.3	1000	$-$ 0.010	1.0	90.7	1000
4	FPAFT-df=9	0.001	0.1	93.4	1000	0.006	0.6	95.2	1000	0.003	1.1	93.5	1000	$-$ 0.010	1.6	89.3	1000
5	FPAFT-df=9	0.004	0.4	94.4	1000	0.002	0.2	93.9	1000	0.002	0.4	94.3	1000	$-$ 0.008	2.2	91.9	1000

**Table 4:** Bias, percentage bias, and coverage of estimates of log($-$log(S(t))) when $X=0$ and $\beta=-0.5$

Time	Model	Scenario 1				Scenario 2				Scenario 3				Scenario 4			
		Bias	$\%$ Bias	Cov.	$\#$ Conv.	Bias	$\%$ Bias	Cov.	$\#$ Conv.	Bias	$\%$ Bias	Cov.	$\#$ Conv.	Bias	$\%$ Bias	Cov.	$\#$ Conv.
1	Weibull	0.586	$-$ 25.4	0.0	1000	$-$ 0.007	.	94.6	1000	0.092	$-$ 8.8	59.8	1000	$-$ 0.008	0.3	94.7	1000
2	Weibull	$-$ 0.018	4.6	92.6	1000	$-$ 0.142	$-$ 22.3	13.6	1000	0.167	$-$ 32.5	9.0	1000	$-$ 0.006	0.4	96.3	1000
3	Weibull	$-$ 0.224	$-$ 38.2	0.7	1000	$-$ 0.086	$-$ 9.8	55.4	1000	0.103	$-$ 107.4	42.3	1000	$-$ 0.004	0.4	96.3	1000
4	Weibull	$-$ 0.015	$-$ 1.6	87.4	1000	0.005	0.5	92.7	1000	$-$ 0.010	$-$ 3.7	96.3	1000	$-$ 0.003	0.5	96.4	1000
5	Weibull	0.256	23.9	1.1	1000	0.081	7.5	63.9	1000	$-$ 0.086	$-$ 15.9	62.9	1000	$-$ 0.002	0.5	96.4	1000
1	Gamma	0.187	$-$ 8.1	45.5	1000	$-$ 0.013	.	94.5	1000	0.064	$-$ 6.1	75.7	1000	$-$ 0.009	0.4	94.6	1000
2	Gamma	0.031	$-$ 8.0	92.1	1000	$-$ 0.143	$-$ 22.5	13.4	1000	0.119	$-$ 23.1	28.7	1000	$-$ 0.005	0.3	96.1	1000
3	Gamma	$-$ 0.169	$-$ 28.8	2.8	1000	$-$ 0.081	$-$ 9.3	59.9	1000	0.077	$-$ 80.3	63.0	1000	$-$ 0.003	0.3	95.7	1000
4	Gamma	$-$ 0.039	$-$ 4.2	82.9	1000	0.016	1.6	91.1	1000	0.000	0.0	95.8	1000	$-$ 0.003	0.5	95.9	1000
5	Gamma	0.132	12.3	23.4	1000	0.097	9.0	57.6	1000	$-$ 0.032	$-$ 5.9	91.2	1000	$-$ 0.002	0.5	96.3	1000
1	GenF	0.007	$-$ 0.3	92.4	984	0.000	.	65.9	686	$-$ 0.004	0.4	18.8	196	$-$ 0.022	1.0	81.5	914
2	GenF	0.004	$-$ 1.0	95.1	993	$-$ 0.025	$-$ 3.9	63.9	692	0.046	$-$ 8.9	25.2	293	$-$ 0.016	1.1	82.4	915
3	GenF	$-$ 0.052	$-$ 8.9	76.3	993	$-$ 0.013	$-$ 1.5	65.0	692	0.027	$-$ 28.2	33.8	365	$-$ 0.010	1.0	82.4	915
4	GenF	$-$ 0.002	$-$ 0.2	93.0	993	0.015	1.5	63.8	692	$-$ 0.002	$-$ 0.7	38.8	404	$-$ 0.004	0.6	81.9	915
5	GenF	0.069	6.4	64.5	993	0.030	2.8	60.4	692	$-$ 0.016	$-$ 3.0	38.4	406	0.000	0.0	82.3	922
1	FPAFT-df=2	0.171	$-$ 7.4	54.0	1000	$-$ 0.039	.	87.3	1000	0.021	$-$ 2.0	92.8	1000	$-$ 0.012	0.5	95.2	1000
2	FPAFT-df=2	0.131	$-$ 33.8	26.0	1000	$-$ 0.147	$-$ 23.1	12.9	1000	0.121	$-$ 23.5	32.2	1000	$-$ 0.005	0.3	96.3	1000
3	FPAFT-df=2	$-$ 0.125	$-$ 21.3	17.5	1000	$-$ 0.070	$-$ 8.0	67.8	1000	0.090	$-$ 93.8	52.6	1000	$-$ 0.003	0.3	96.1	1000
4	FPAFT-df=2	$-$ 0.045	$-$ 4.9	78.6	1000	0.037	3.7	86.5	1000	0.004	1.5	96.2	1000	$-$ 0.002	0.3	96.4	1000
5	FPAFT-df=2	0.101	9.4	45.7	1000	0.125	11.6	37.6	1000	$-$ 0.049	$-$ 9.1	85.7	1000	$-$ 0.002	0.5	96.4	1000
1	FPAFT-df=3	$-$ 0.104	4.5	85.1	1000	0.066	.	73.0	1000	0.031	$-$ 3.0	93.2	1000	$-$ 0.009	0.4	94.2	1000
2	FPAFT-df=3	0.140	$-$ 36.2	22.0	1000	$-$ 0.079	$-$ 12.4	60.6	1000	0.046	$-$ 8.9	84.7	1000	$-$ 0.004	0.3	96.3	1000
3	FPAFT-df=3	$-$ 0.050	$-$ 8.5	81.5	1000	$-$ 0.052	$-$ 6.0	78.5	1000	0.030	$-$ 31.3	90.5	1000	$-$ 0.004	0.4	95.8	1000
4	FPAFT-df=3	$-$ 0.032	$-$ 3.5	87.8	1000	0.013	1.3	91.9	1000	$-$ 0.019	$-$ 7.1	93.4	1000	$-$ 0.003	0.5	95.9	1000
5	FPAFT-df=3	0.039	3.6	84.3	1000	0.068	6.3	73.9	1000	$-$ 0.037	$-$ 6.8	88.9	1000	$-$ 0.002	0.5	96.3	1000
1	FPAFT-df=4	0.005	$-$ 0.2	94.5	1000	0.072	.	64.2	1000	0.013	$-$ 1.2	94.5	1000	$-$ 0.007	0.3	94.3	1000
2	FPAFT-df=4	0.074	$-$ 19.1	67.0	1000	$-$ 0.017	$-$ 2.7	93.6	1000	0.054	$-$ 10.5	82.1	1000	$-$ 0.006	0.4	96.2	1000
3	FPAFT-df=4	$-$ 0.022	$-$ 3.8	92.8	1000	$-$ 0.024	$-$ 2.7	91.4	1000	0.041	$-$ 42.8	88.3	1000	$-$ 0.004	0.4	95.9	1000
4	FPAFT-df=4	0.017	$-$ 1.8	93.9	1000	$-$ 0.000	0.0	94.6	1000	$-$ 0.011	$-$ 4.1	94.7	1000	$-$ 0.003	0.5	95.3	1000
5	FPAFT-df=4	0.026	2.4	89.7	1000	0.019	1.8	90.7	1000	$-$ 0.033	$-$ 6.1	90.2	1000	$-$ 0.002	0.5	96.3	1000
1	FPAFT-df=5	0.004	$-$ 0.2	94.4	1000	0.044	.	82.2	1000	0.024	$-$ 2.3	94.0	1000	$-$ 0.007	0.3	94.6	1000
2	FPAFT-df=5	0.023	$-$ 5.9	90.4	1000	$-$ 0.007	$-$ 1.1	94.2	1000	0.037	$-$ 7.2	88.0	1000	$-$ 0.006	0.4	95.7	1000
3	FPAFT-df=5	$-$ 0.012	$-$ 2.0	94.3	1000	$-$ 0.017	$-$ 1.9	93.0	1000	0.059	$-$ 61.5	80.2	1000	$-$ 0.005	0.5	95.4	1000
4	FPAFT-df=5	$-$ 0.006	$-$ 0.7	94.9	1000	$-$ 0.000	0.0	94.9	1000	0.008	3.0	93.6	1000	$-$ 0.004	0.6	94.9	1000
5	FPAFT-df=5	0.024	2.2	90.6	1000	0.011	1.0	93.0	1000	$-$ 0.023	$-$ 4.3	92.3	1000	$-$ 0.003	0.8	95.9	1000
1	FPAFT-df=6	$-$ 0.007	0.3	94.7	1000	0.018	.	91.6	1000	0.026	$-$ 2.5	92.7	1000	$-$ 0.007	0.3	94.7	1000
2	FPAFT-df=6	$-$ 0.001	0.3	94.0	1000	$-$ 0.001	$-$ 0.2	94.0	1000	0.026	$-$ 5.1	91.7	1000	$-$ 0.006	0.4	95.5	1000
3	FPAFT-df=6	$-$ 0.009	$-$ 1.5	94.2	1000	$-$ 0.009	$-$ 1.0	94.3	1000	0.064	$-$ 66.7	76.4	1000	$-$ 0.005	0.5	95.0	1000
4	FPAFT-df=6	0.001	0.1	94.5	1000	0.002	0.2	94.1	1000	0.022	8.2	91.3	1000	$-$ 0.004	0.6	94.0	1000
5	FPAFT-df=6	0.025	2.3	90.7	1000	0.008	0.7	93.7	1000	$-$ 0.017	$-$ 3.1	92.8	1000	$-$ 0.003	0.8	94.5	1000
1	FPAFT-df=7	$-$ 0.006	0.3	94.4	1000	0.004	.	93.8	1000	0.020	$-$ 1.9	93.4	1000	$-$ 0.007	0.3	94.9	1000
2	FPAFT-df=7	$-$ 0.008	2.1	94.4	1000	0.001	0.2	93.9	1000	0.025	$-$ 4.9	90.6	1000	$-$ 0.006	0.4	94.9	1000
3	FPAFT-df=7	$-$ 0.009	$-$ 1.5	94.5	1000	$-$ 0.006	$-$ 0.7	94.9	1000	0.059	$-$ 61.5	77.1	1000	$-$ 0.005	0.5	93.1	1000
4	FPAFT-df=7	0.006	0.7	93.5	1000	0.003	0.3	93.5	1000	0.031	11.5	88.9	1000	$-$ 0.003	0.5	92.3	1000
5	FPAFT-df=7	0.026	2.4	90.4	1000	0.006	0.6	93.1	1000	$-$ 0.013	$-$ 2.4	92.9	1000	$-$ 0.003	0.8	92.0	1000
1	FPAFT-df=8	$-$ 0.012	0.5	94.1	1000	$-$ 0.002	.	92.7	1000	0.020	$-$ 1.9	93.5	1000	$-$ 0.006	0.3	94.2	1000
2	FPAFT-df=8	$-$ 0.009	2.3	95.3	1000	0.000	0.0	93.3	1000	0.030	$-$ 5.8	89.7	1000	$-$ 0.004	0.3	93.0	1000
3	FPAFT-df=8	$-$ 0.010	$-$ 1.7	93.2	1000	$-$ 0.003	$-$ 0.3	94.0	1000	0.050	$-$ 52.1	81.9	1000	$-$ 0.002	0.2	92.5	1000
4	FPAFT-df=8	0.009	1.0	92.9	1000	0.004	0.4	93.7	1000	0.037	13.7	85.8	1000	$-$ 0.001	0.2	91.1	1000
5	FPAFT-df=8	0.026	2.4	90.3	1000	0.006	0.6	93.1	1000	$-$ 0.011	$-$ 2.0	91.7	1000	$-$ 0.000	0.0	89.6	1000
1	FPAFT-df=9	$-$ 0.014	0.6	94.8	1000	$-$ 0.002	.	93.9	1000	0.020	$-$ 1.9	93.3	1000	$-$ 0.004	0.2	93.6	1000
2	FPAFT-df=9	$-$ 0.008	2.1	94.4	1000	$-$ 0.000	0.0	93.5	1000	0.030	$-$ 5.8	89.3	1000	$-$ 0.000	0.0	91.7	1000
3	FPAFT-df=9	$-$ 0.012	$-$ 2.0	92.1	1000	$-$ 0.002	$-$ 0.2	94.4	1000	0.041	$-$ 42.8	83.7	1000	0.001	$-$ 0.1	90.1	1000
4	FPAFT-df=9	0.011	1.2	92.6	1000	0.005	0.5	93.0	1000	0.041	15.2	82.2	1000	0.002	$-$ 0.3	87.8	1000
5	FPAFT-df=9	0.027	2.5	90.0	1000	0.006	0.6	92.9	1000	$-$ 0.009	$-$ 1.7	90.9	1000	0.004	$-$ 1.1	86.9	1000

**Table 5:** Bias, percentage bias, and coverage of estimates of log($-$log(S(t))) when $X=1$ and $\beta=0.5$

Time	Model	Scenario 1				Scenario 2				Scenario 3				Scenario 4			
		Bias	$\%$ Bias	Cov.	$\#$ Conv.	Bias	$\%$ Bias	Cov.	$\#$ Conv.	Bias	$\%$ Bias	Cov.	$\#$ Conv.	Bias	$\%$ Bias	Cov.	$\#$ Conv.
1	Weibull	0.606	$-$ 16.7	0.0	1000	0.129	$-$ 26.4	27.0	1000	$-$ 0.055	4.0	84.9	1000	$-$ 0.012	0.4	94.6	1000
2	Weibull	0.303	$-$ 17.1	0.2	1000	$-$ 0.029	$-$ 15.1	91.1	1000	0.046	$-$ 5.1	87.1	1000	$-$ 0.009	0.4	94.8	1000
3	Weibull	0.079	$-$ 12.2	66.6	1000	$-$ 0.095	$-$ 16.9	47.8	1000	0.072	$-$ 12.1	74.5	1000	$-$ 0.007	0.4	95.0	1000
4	Weibull	$-$ 0.045	$-$ 37.8	85.4	1000	$-$ 0.079	$-$ 10.3	62.9	1000	0.047	$-$ 14.2	86.3	1000	$-$ 0.006	0.5	96.2	1000
5	Weibull	$-$ 0.035	$-$ 5.8	90.6	1000	$-$ 0.025	$-$ 2.8	92.8	1000	$-$ 0.014	16.9	95.0	1000	$-$ 0.005	0.5	96.0	1000
1	Gamma	$-$ 0.128	3.5	82.7	1000	0.107	$-$ 21.9	42.0	1000	$-$ 0.040	2.9	83.4	929	$-$ 0.011	0.4	93.8	1000
2	Gamma	0.182	$-$ 10.3	34.3	1000	$-$ 0.041	$-$ 21.3	85.8	1000	0.007	$-$ 0.8	88.0	929	$-$ 0.006	0.3	95.0	1000
3	Gamma	0.089	$-$ 13.8	63.4	1000	$-$ 0.090	$-$ 16.0	52.6	1000	0.030	$-$ 5.0	83.3	929	$-$ 0.005	0.3	95.0	1000
4	Gamma	$-$ 0.038	$-$ 31.9	89.9	1000	$-$ 0.057	$-$ 7.5	78.0	1000	0.024	$-$ 7.3	85.3	929	$-$ 0.006	0.5	95.8	1000
5	Gamma	$-$ 0.077	$-$ 12.7	64.2	1000	0.015	1.7	94.0	1000	$-$ 0.003	3.6	88.5	929	$-$ 0.005	0.5	95.7	1000
1	GenF	0.053	$-$ 1.5	89.9	985	0.033	$-$ 6.8	59.8	661	$-$ 0.048	3.5	18.5	222	$-$ 0.021	0.7	67.2	761
2	GenF	$-$ 0.018	1.0	92.7	992	0.001	0.5	68.1	713	0.005	$-$ 0.6	28.6	320	$-$ 0.014	0.7	70.4	792
3	GenF	$-$ 0.012	1.9	93.5	995	$-$ 0.015	$-$ 2.7	67.5	713	0.030	$-$ 5.0	32.7	361	$-$ 0.006	0.4	70.9	797
4	GenF	0.006	5.0	95.4	996	$-$ 0.019	$-$ 2.5	66.8	713	0.025	$-$ 7.6	36.3	394	$-$ 0.003	0.2	71.6	803
5	GenF	$-$ 0.039	$-$ 6.4	85.0	996	$-$ 0.002	$-$ 0.2	67.6	713	$-$ 0.003	3.6	54.1	606	$-$ 0.001	0.1	74.6	840
1	FPAFT-df=2	$-$ 0.380	10.5	47.1	1000	0.080	$-$ 16.4	64.1	1000	$-$ 0.118	8.5	58.2	1000	$-$ 0.021	0.7	94.4	1000
2	FPAFT-df=2	0.103	$-$ 5.8	67.5	1000	$-$ 0.046	$-$ 23.9	83.9	1000	$-$ 0.017	1.9	92.7	1000	$-$ 0.011	0.5	94.6	1000
3	FPAFT-df=2	0.086	$-$ 13.3	67.1	1000	$-$ 0.082	$-$ 14.6	59.0	1000	0.030	$-$ 5.0	90.5	1000	$-$ 0.007	0.4	94.7	1000
4	FPAFT-df=2	$-$ 0.033	$-$ 27.7	92.4	1000	$-$ 0.042	$-$ 5.5	85.4	1000	0.029	$-$ 8.8	90.7	1000	$-$ 0.006	0.5	95.7	1000
5	FPAFT-df=2	$-$ 0.101	$-$ 16.7	38.8	1000	0.033	3.8	89.1	1000	$-$ 0.008	9.6	95.1	1000	$-$ 0.005	0.5	95.7	1000
1	FPAFT-df=3	0.010	$-$ 0.3	92.1	1000	0.059	$-$ 12.1	79.8	1000	$-$ 0.002	0.1	96.3	1000	$-$ 0.019	0.7	93.9	1000
2	FPAFT-df=3	$-$ 0.002	0.1	95.4	1000	$-$ 0.022	$-$ 11.4	95.2	1000	$-$ 0.003	0.3	97.0	1000	$-$ 0.009	0.4	93.8	1000
3	FPAFT-df=3	$-$ 0.015	2.3	94.4	1000	$-$ 0.083	$-$ 14.7	53.5	1000	$-$ 0.000	0.0	95.7	1000	$-$ 0.007	0.4	94.9	1000
4	FPAFT-df=3	0.005	4.2	94.7	1000	$-$ 0.085	$-$ 11.1	52.5	1000	0.002	$-$ 0.6	95.3	1000	$-$ 0.006	0.5	96.2	1000
5	FPAFT-df=3	$-$ 0.005	$-$ 0.8	95.3	1000	$-$ 0.052	$-$ 5.9	77.2	1000	$-$ 0.002	2.4	94.6	1000	$-$ 0.005	0.5	94.9	1000
1	FPAFT-df=4	0.007	$-$ 0.2	93.3	1000	$-$ 0.012	2.5	95.4	1000	$-$ 0.009	0.6	95.6	1000	$-$ 0.018	0.6	94.6	1000
2	FPAFT-df=4	$-$ 0.007	0.4	95.0	1000	0.006	3.1	92.8	1000	$-$ 0.008	0.9	93.8	1000	$-$ 0.008	0.4	94.8	1000
3	FPAFT-df=4	$-$ 0.006	0.9	95.1	1000	0.002	0.4	94.8	1000	0.004	$-$ 0.7	93.7	1000	$-$ 0.006	0.4	95.3	1000
4	FPAFT-df=4	0.003	2.5	95.2	1000	$-$ 0.009	$-$ 1.2	94.7	1000	0.006	$-$ 1.8	94.2	1000	$-$ 0.006	0.5	94.6	1000
5	FPAFT-df=4	$-$ 0.006	$-$ 1.0	94.8	1000	$-$ 0.005	$-$ 0.6	94.7	1000	$-$ 0.002	2.4	94.6	1000	$-$ 0.005	0.5	95.4	1000
1	FPAFT-df=5	0.002	$-$ 0.1	94.1	1000	$-$ 0.013	2.7	95.3	1000	$-$ 0.007	0.5	94.2	1000	$-$ 0.016	0.6	94.2	1000
2	FPAFT-df=5	$-$ 0.008	0.5	95.3	1000	0.005	2.6	94.1	1000	$-$ 0.005	0.6	95.2	1000	$-$ 0.006	0.3	93.6	1000
3	FPAFT-df=5	$-$ 0.009	1.4	94.7	1000	0.002	0.4	94.3	1000	$-$ 0.008	1.3	95.1	1000	$-$ 0.006	0.4	94.1	1000
4	FPAFT-df=5	0.004	3.4	94.8	1000	$-$ 0.005	$-$ 0.7	95.6	1000	$-$ 0.004	1.2	95.0	1000	$-$ 0.005	0.4	94.2	1000
5	FPAFT-df=5	$-$ 0.007	$-$ 1.2	94.8	1000	$-$ 0.001	$-$ 0.1	95.4	1000	$-$ 0.002	2.4	94.3	1000	$-$ 0.003	0.3	94.8	1000
1	FPAFT-df=6	$-$ 0.003	0.1	95.0	1000	$-$ 0.003	0.6	94.9	1000	$-$ 0.006	0.4	94.4	1000	$-$ 0.015	0.5	93.2	1000
2	FPAFT-df=6	$-$ 0.011	0.6	94.4	1000	0.002	1.0	94.0	1000	$-$ 0.005	0.6	95.1	1000	$-$ 0.007	0.3	93.5	1000
3	FPAFT-df=6	$-$ 0.003	0.5	93.8	1000	0.002	0.4	94.9	1000	$-$ 0.005	0.8	95.3	1000	$-$ 0.006	0.4	93.0	1000
4	FPAFT-df=6	$-$ 0.001	$-$ 0.8	93.6	1000	$-$ 0.000	0.0	95.5	1000	$-$ 0.006	1.8	94.9	1000	$-$ 0.005	0.4	92.5	1000
5	FPAFT-df=6	$-$ 0.001	$-$ 0.2	94.3	1000	0.003	0.3	95.4	1000	$-$ 0.002	2.4	94.7	1000	$-$ 0.003	0.3	93.4	1000
1	FPAFT-df=7	$-$ 0.006	0.2	94.3	1000	$-$ 0.001	0.2	93.7	1000	$-$ 0.005	0.4	94.6	1000	$-$ 0.014	0.5	93.1	1000
2	FPAFT-df=7	$-$ 0.011	0.6	94.2	1000	0.002	1.0	93.3	1000	$-$ 0.004	0.4	94.9	1000	$-$ 0.005	0.2	92.7	1000
3	FPAFT-df=7	$-$ 0.002	0.3	94.5	1000	0.000	0.0	93.5	1000	$-$ 0.003	0.5	94.1	1000	$-$ 0.005	0.3	91.4	1000
4	FPAFT-df=7	$-$ 0.001	$-$ 0.8	93.9	1000	$-$ 0.001	$-$ 0.1	95.2	1000	$-$ 0.004	1.2	95.0	1000	$-$ 0.004	0.3	91.1	1000
5	FPAFT-df=7	0.001	0.2	94.3	1000	0.003	0.3	95.2	1000	$-$ 0.001	1.2	94.5	1000	$-$ 0.002	0.2	91.0	1000
1	FPAFT-df=8	$-$ 0.009	0.2	95.3	1000	$-$ 0.002	0.4	94.4	1000	$-$ 0.005	0.4	93.7	1000	$-$ 0.011	0.4	92.2	1000
2	FPAFT-df=8	$-$ 0.010	0.6	93.7	1000	0.000	0.0	92.9	1000	$-$ 0.004	0.4	94.6	1000	$-$ 0.003	0.1	91.2	1000
3	FPAFT-df=8	$-$ 0.002	0.3	94.0	1000	$-$ 0.000	0.0	94.3	1000	$-$ 0.003	0.5	93.8	1000	$-$ 0.003	0.2	90.6	1000
4	FPAFT-df=8	0.000	0.0	92.7	1000	$-$ 0.002	$-$ 0.3	94.4	1000	$-$ 0.003	0.9	94.1	1000	$-$ 0.002	0.2	89.5	1000
5	FPAFT-df=8	0.001	0.2	93.6	1000	0.002	0.2	95.0	1000	$-$ 0.001	1.2	92.4	1000	$-$ 0.000	0.0	88.5	1000
1	FPAFT-df=9	$-$ 0.012	0.3	94.9	1000	$-$ 0.001	0.2	92.4	1000	$-$ 0.005	0.4	94.5	1000	$-$ 0.007	0.2	92.4	1000
2	FPAFT-df=9	$-$ 0.009	0.5	93.4	1000	0.001	0.5	91.6	1000	$-$ 0.004	0.4	94.3	1000	$-$ 0.001	0.0	90.3	1000
3	FPAFT-df=9	$-$ 0.002	0.3	92.6	1000	0.001	0.2	93.3	1000	$-$ 0.003	0.5	93.1	1000	$-$ 0.000	0.0	90.1	1000
4	FPAFT-df=9	0.000	0.0	93.7	1000	$-$ 0.002	$-$ 0.3	94.6	1000	$-$ 0.003	0.9	93.2	1000	0.001	$-$ 0.1	88.0	1000
5	FPAFT-df=9	0.000	0.0	93.7	1000	0.002	0.2	94.7	1000	$-$ 0.001	1.2	92.8	1000	0.002	$-$ 0.2	88.5	1000

**Table 6:** Bias, percentage bias, and coverage of estimates of log($-$log(S(t))) when $X=1$ and $\beta=-0.5$

Time	Model	Scenario 1				Scenario 2				Scenario 3				Scenario 4			
		Bias	$\%$ Bias	Cov.	$\#$ Conv.	Bias	$\%$ Bias	Cov.	$\#$ Conv.	Bias	$\%$ Bias	Cov.	$\#$ Conv.	Bias	$\%$ Bias	Cov.	$\#$ Conv.
1	Weibull	0.042	$-$ 4.6	87.5	1000	$-$ 0.156	$-$ 32.5	9.7	1000	0.153	$-$ 22.6	18.2	1000	$-$ 0.007	0.4	94.2	1000
2	Weibull	$-$ 0.301	$-$ 40.9	0.1	1000	$-$ 0.089	$-$ 9.7	52.1	1000	0.062	314.2	73.7	1000	$-$ 0.004	0.5	94.8	1000
3	Weibull	0.137	12.9	29.1	1000	0.047	4.4	79.1	1000	$-$ 0.092	$-$ 17.4	48.9	1000	$-$ 0.002	0.5	95.7	1000
4	Weibull	0.450	34.7	0.0	1000	0.144	12.2	24.7	1000	$-$ 0.085	$-$ 11.0	58.3	1000	$-$ 0.001	2.6	95.0	1000
5	Weibull	0.657	43.5	0.0	1000	0.216	17.0	3.9	1000	$-$ 0.029	$-$ 3.2	93.0	1000	0.000	0.0	95.1	1000
1	Gamma	0.111	$-$ 12.1	55.0	1000	$-$ 0.163	$-$ 34.0	7.4	1000	0.044	$-$ 6.5	86.5	1000	$-$ 0.005	0.3	94.7	1000
2	Gamma	$-$ 0.176	$-$ 23.9	2.9	1000	$-$ 0.087	$-$ 9.5	53.4	1000	$-$ 0.024	$-$ 121.6	92.9	1000	$-$ 0.003	0.3	95.2	1000
3	Gamma	0.111	10.4	34.7	1000	0.058	5.4	74.8	1000	$-$ 0.118	$-$ 22.3	31.1	1000	$-$ 0.002	0.5	95.0	1000
4	Gamma	0.258	19.9	0.7	1000	0.162	13.7	21.6	1000	$-$ 0.040	$-$ 5.2	85.2	1000	$-$ 0.001	2.6	94.9	1000
5	Gamma	0.310	20.5	0.3	1000	0.241	19.0	3.5	1000	0.089	9.8	66.4	1000	$-$ 0.000	0.0	94.5	1000
1	GenF	$-$ 0.026	2.8	91.8	991	$-$ 0.013	$-$ 2.7	65.6	692	0.041	$-$ 6.1	23.0	257	$-$ 0.009	0.5	80.7	914
2	GenF	$-$ 0.059	$-$ 8.0	67.4	993	$-$ 0.004	$-$ 0.4	65.3	692	0.019	96.3	36.6	384	0.001	$-$ 0.1	81.2	914
3	GenF	0.066	6.2	64.9	993	0.029	2.7	61.8	692	$-$ 0.013	$-$ 2.5	38.5	407	0.009	$-$ 2.3	81.5	918
4	GenF	0.066	5.1	69.0	993	0.034	2.9	61.2	692	0.007	0.9	38.8	407	0.007	$-$ 17.9	82.0	922
5	GenF	$-$ 0.001	$-$ 0.1	92.8	993	0.026	2.0	63.4	692	0.034	3.7	37.6	407	$-$ 0.003	$-$ 1.3	82.1	923
1	FPAFT-df=2	0.105	$-$ 11.4	58.9	1000	$-$ 0.168	$-$ 35.0	7.4	1000	0.066	$-$ 9.7	75.7	1000	$-$ 0.005	0.3	94.9	1000
2	FPAFT-df=2	$-$ 0.210	$-$ 28.5	0.2	1000	$-$ 0.067	$-$ 7.3	65.9	1000	0.027	136.8	90.2	1000	$-$ 0.002	0.2	95.2	1000
3	FPAFT-df=2	0.025	2.3	84.4	1000	0.091	8.5	56.2	1000	$-$ 0.088	$-$ 16.6	53.9	1000	$-$ 0.001	0.3	95.2	1000
4	FPAFT-df=2	0.174	13.4	23.7	1000	0.203	17.2	7.8	1000	$-$ 0.051	$-$ 6.6	82.4	1000	$-$ 0.001	2.6	94.8	1000
5	FPAFT-df=2	0.254	16.8	12.4	1000	0.288	22.7	0.6	1000	0.028	3.1	90.5	1000	$-$ 0.000	0.0	94.8	1000
1	FPAFT-df=3	$-$ 0.005	0.5	92.3	1000	$-$ 0.130	$-$ 27.1	14.3	1000	0.008	$-$ 1.2	96.3	1000	$-$ 0.003	0.2	94.7	1000
2	FPAFT-df=3	$-$ 0.124	$-$ 16.9	8.1	1000	$-$ 0.102	$-$ 11.2	37.7	1000	$-$ 0.029	$-$ 147.0	90.7	1000	$-$ 0.004	0.5	95.2	1000
3	FPAFT-df=3	$-$ 0.002	$-$ 0.2	92.2	1000	$-$ 0.003	$-$ 0.3	87.9	1000	$-$ 0.086	$-$ 16.3	55.4	1000	$-$ 0.003	0.8	95.1	1000
4	FPAFT-df=3	0.046	3.5	85.7	1000	0.065	5.5	76.2	1000	$-$ 0.001	$-$ 0.1	93.5	1000	$-$ 0.001	2.6	95.0	1000
5	FPAFT-df=3	0.047	3.1	87.1	1000	0.116	9.1	59.2	1000	0.114	12.5	44.0	1000	$-$ 0.000	0.0	95.1	1000
1	FPAFT-df=4	$-$ 0.061	6.6	81.6	1000	$-$ 0.034	$-$ 7.1	90.6	1000	$-$ 0.000	0.0	93.7	1000	$-$ 0.005	0.3	94.5	1000
2	FPAFT-df=4	$-$ 0.070	$-$ 9.5	56.2	1000	$-$ 0.047	$-$ 5.1	76.9	1000	$-$ 0.025	$-$ 126.7	93.5	1000	$-$ 0.003	0.3	95.4	1000
3	FPAFT-df=4	0.007	0.7	94.3	1000	$-$ 0.009	$-$ 0.8	92.3	1000	$-$ 0.087	$-$ 16.4	55.4	1000	$-$ 0.002	0.5	95.0	1000
4	FPAFT-df=4	0.015	1.2	94.3	1000	0.013	1.1	93.0	1000	$-$ 0.008	$-$ 1.0	91.9	1000	$-$ 0.001	2.6	94.4	1000
5	FPAFT-df=4	$-$ 0.014	$-$ 0.9	92.3	1000	0.027	2.1	91.3	1000	0.102	11.2	55.1	1000	$-$ 0.000	0.0	95.0	1000
1	FPAFT-df=5	$-$ 0.045	4.9	87.9	1000	$-$ 0.013	$-$ 2.7	96.2	1000	$-$ 0.026	3.8	90.6	1000	$-$ 0.004	0.2	94.5	1000
2	FPAFT-df=5	$-$ 0.044	$-$ 6.0	82.9	1000	$-$ 0.029	$-$ 3.2	88.6	1000	$-$ 0.018	$-$ 91.2	94.9	1000	$-$ 0.002	0.2	94.6	1000
3	FPAFT-df=5	0.015	1.4	93.7	1000	$-$ 0.005	$-$ 0.5	93.8	1000	$-$ 0.085	$-$ 16.1	53.7	1000	$-$ 0.002	0.5	94.3	1000
4	FPAFT-df=5	0.005	0.4	95.7	1000	0.007	0.6	93.6	1000	$-$ 0.020	$-$ 2.6	90.2	1000	$-$ 0.001	2.6	94.2	1000
5	FPAFT-df=5	$-$ 0.039	$-$ 2.6	88.0	1000	0.014	1.1	93.4	1000	0.079	8.7	70.2	1000	0.000	0.0	94.3	1000
1	FPAFT-df=6	$-$ 0.018	2.0	95.1	1000	$-$ 0.002	$-$ 0.4	95.0	1000	$-$ 0.023	3.4	92.9	1000	$-$ 0.004	0.2	94.4	1000
2	FPAFT-df=6	$-$ 0.029	$-$ 3.9	90.1	1000	$-$ 0.016	$-$ 1.8	93.9	1000	$-$ 0.016	$-$ 81.1	94.3	1000	$-$ 0.002	0.2	94.1	1000
3	FPAFT-df=6	0.021	2.0	92.8	1000	$-$ 0.001	$-$ 0.1	94.7	1000	$-$ 0.079	$-$ 14.9	58.4	1000	$-$ 0.002	0.5	93.0	1000
4	FPAFT-df=6	0.001	0.1	96.0	1000	0.004	0.3	94.4	1000	$-$ 0.027	$-$ 3.5	88.6	1000	$-$ 0.001	2.6	92.6	1000
5	FPAFT-df=6	$-$ 0.050	$-$ 3.3	86.1	1000	0.006	0.5	94.3	1000	0.064	7.0	77.7	1000	$-$ 0.000	0.0	93.8	1000
1	FPAFT-df=7	$-$ 0.004	0.4	94.5	1000	0.003	0.6	93.8	1000	$-$ 0.020	3.0	92.6	1000	$-$ 0.004	0.2	93.4	1000
2	FPAFT-df=7	$-$ 0.019	$-$ 2.6	93.6	1000	$-$ 0.007	$-$ 0.8	95.3	1000	$-$ 0.018	$-$ 91.2	91.7	1000	$-$ 0.002	0.2	93.2	1000
3	FPAFT-df=7	0.026	2.4	91.0	1000	0.003	0.3	94.8	1000	$-$ 0.072	$-$ 13.6	62.3	1000	$-$ 0.003	0.8	90.9	1000
4	FPAFT-df=7	$-$ 0.001	$-$ 0.1	96.2	1000	0.004	0.3	94.2	1000	$-$ 0.030	$-$ 3.9	86.4	1000	$-$ 0.001	2.6	92.9	1000
5	FPAFT-df=7	$-$ 0.057	$-$ 3.8	84.7	1000	0.002	0.2	94.2	1000	0.053	5.8	82.6	1000	$-$ 0.000	0.0	93.8	1000
1	FPAFT-df=8	0.001	$-$ 0.1	94.0	1000	0.004	0.8	93.5	1000	$-$ 0.022	3.2	92.0	1000	$-$ 0.006	0.4	92.2	1000
2	FPAFT-df=8	$-$ 0.013	$-$ 1.8	94.5	1000	$-$ 0.003	$-$ 0.3	95.4	1000	$-$ 0.022	$-$ 111.5	89.5	1000	$-$ 0.005	0.6	91.0	1000
3	FPAFT-df=8	0.029	2.7	89.8	1000	0.005	0.5	94.6	1000	$-$ 0.066	$-$ 12.5	66.4	1000	$-$ 0.005	1.3	88.7	1000
4	FPAFT-df=8	$-$ 0.001	$-$ 0.1	96.1	1000	0.004	0.3	94.4	1000	$-$ 0.031	$-$ 4.0	85.2	1000	$-$ 0.003	7.7	89.8	1000
5	FPAFT-df=8	$-$ 0.060	$-$ 4.0	83.5	1000	0.000	0.0	93.9	1000	0.046	5.0	85.6	1000	$-$ 0.002	$-$ 0.9	92.4	1000
1	FPAFT-df=9	0.002	$-$ 0.2	93.7	1000	0.004	0.8	92.5	1000	$-$ 0.023	3.4	91.8	1000	$-$ 0.009	0.5	91.8	1000
2	FPAFT-df=9	$-$ 0.010	$-$ 1.4	94.6	1000	0.000	0.0	95.5	1000	$-$ 0.025	$-$ 126.7	86.6	1000	$-$ 0.008	0.9	89.6	1000
3	FPAFT-df=9	0.030	2.8	89.4	1000	0.006	0.6	95.1	1000	$-$ 0.061	$-$ 11.5	71.9	1000	$-$ 0.008	2.1	85.7	1000
4	FPAFT-df=9	$-$ 0.001	$-$ 0.1	96.2	1000	0.003	0.3	94.8	1000	$-$ 0.031	$-$ 4.0	84.5	1000	$-$ 0.006	15.4	89.3	1000
5	FPAFT-df=9	$-$ 0.062	$-$ 4.1	84.0	1000	$-$ 0.001	$-$ 0.1	94.8	1000	0.041	4.5	85.1	1000	$-$ 0.004	$-$ 1.7	91.7	1000

From Table 2, looking at Scenarios 1–3, the Weibull AFT model gives substantial bias in estimates of the log acceleration factor, and poor coverage probabilities. Similarly, but to a lesser extent, the generalized gamma also indicates some bias and poor coverage, but in addition an important proportion of models, 316 out of 1000, failed to converge in Scenario 3 when $\beta=0.5$. The generalized F model performed particularly poorly as a substantial proportional in most scenarios failed to converge; therefore the bias and coverage estimates calculated only on models which converged, should be interpreted with caution. Results based on those that did converge indicate some bias across scenarios, but particularly poor coverage across all scenarios. In all scenarios, the flexible parametric AFT performed well across varying degrees of freedom. In Scenarios 1 to 3, there was a FPAFT with a specific degree of freedom (or multiple), that outperformed the Weibull, generalized gamma, and generalized F, both in terms of less bias and the coverage probabilities being closer to 95$\%$. When there was bias in specific degrees of freedom, the AIC and BIC indicated a more appropriate well-fitting model, generally with the least bias. In Scenario 4, where the true model was a Weibull (equivalent to FPAFT with df = 1), generally all models estimated the log acceleration factor with minimal bias; however, coverage began to be suboptimal as the degrees of freedom increased in the FPAFT, clearly due to over-fitting. Generally, a flexible parametric AFT model was the best fitting in terms of both AIC and BIC, apart from Scenario 4 where the true Weibull model (which is equivalent to a flexible parametric AFT with 1 degree of freedom). In some settings the generalized F was best fitting; however, this is based only on estimates that converged (e.g., scenario 3 and $\beta=-0.5$, only 397 out of 1000 converged). 

Moving to estimates of survival in Tables 3–6, in Scenarios 1 to 3, the Weibull model produced substantial bias and poor coverage, compared to excellent performance in Scenario 4 when the truth was Weibull. Both the generalized gamma and generalized F models produced varying levels of bias and poor coverage, particularly the generalized F, across all four scenarios. Both suffered from varying levels of lack of convergence, and also the delta method failed to calculate a standard error in some simulations. The flexible parametric AFT model performed well across all four scenarios; however, Scenario 3 posed particular problems in capturing estimates of survival at early time points. Generally, there was at least one degree of freedom which provided generally unbiased estimates of survival in each treatment group, with coverage around 95$\%$. 

## 5. Breast cancer in england and wales

To illustrate the proposed AFT model, we use a data set of 9721 women aged under 50 and diagnosed with breast cancer in England and Wales between 1986 and 1990. Our event of interest is death from any cause, where 2847 events were observed, and we have restricted follow-up to 5 years, leading to 6850 censored at 5 years. We are interested in the effect of deprivation status, which was categorized into least and most deprived groups. We subsequently have a binary covariate of interest, with 0 for the least deprived and 1 for the most deprived group. All models are also adjusted for sex and age at diagnosis. 

We fit Weibull, generalized gamma, generalized F and the proposed AFT models with 2–9 degrees of freedom, and present estimates of the log acceleration factor for the effect of deprivation status, its standard error and associated 95$\%$ confidence interval in Table 7, and also model fit statistics, namely the AIC and BIC. 

**Table 7:** Comparison of parametric AFT models applied to the England and Wales breast cancer data set

Model	Estimate	Std. Err.	95$\%$ CI		AIC	BIC
Weibull	$-$ 0.244	0.038	$-$ 0.318	$-$ 0.170	17 595.51	17 619.32
Gen. Gamma	$-$ 0.274	0.041	$-$ 0.354	$-$ 0.194	17 579.75	17 609.52
Gen. F	$-$ 0.343	0.041	$-$ 0.362	$-$ 0.186	17 518.01	17 553.74
FPAFT df=2	$-$ 0.337	0.044	$-$ 0.424	$-$ 0.250	17 524.72	17 560.44
FPAFT df=3	$-$ 0.303	0.045	$-$ 0.391	$-$ 0.216	17 501.11	17 542.79
FPAFT df=4	$-$ 0.311	0.045	$-$ 0.398	$-$ 0.223	17 500.59	17 548.22
FPAFT df=5	$-$ 0.312	0.043	$-$ 0.397	$-$ 0.227	17 500.11	17 553.70
FPAFT df=6	$-$ 0.306	0.044	$-$ 0.394	$-$ 0.219	17 501.89	17 561.43
FPAFT df=7	$-$ 0.299	0.043	$-$ 0.384	$-$ 0.214	17 504.33	17 569.83
FPAFT df=8	$-$ 0.306	0.044	$-$ 0.391	$-$ 0.220	17 504.99	17 576.44
FPAFT df=9	$-$ 0.302	0.046	$-$ 0.392	$-$ 0.212	17 507.00	17 584.40

Table 7 indicates that the best fitting model, both in terms of lowest AIC and BIC, is a flexible parametric AFT model with 3 degrees of freedom (favoring simpler models). This estimates an acceleration factor of 0.739 (95$\%$ CI 0.676–0.806) for the effect of deprivation status, indicating a patient’s survival time is reduced by 26.1$\%$ (95$\%$ CI 19.4–32.4) by being in the most deprived group, compared to the least deprived. 

The differences in AIC and BIC are substantial between the best fitting model, and those commonly used, namely the Weibull, gamma and F models. We also observe important variation in the estimates of the effect of deprivation status between the FPAFT with df = 3, and the Weibull, gamma and F model estimates. 

**Fig. 3:**
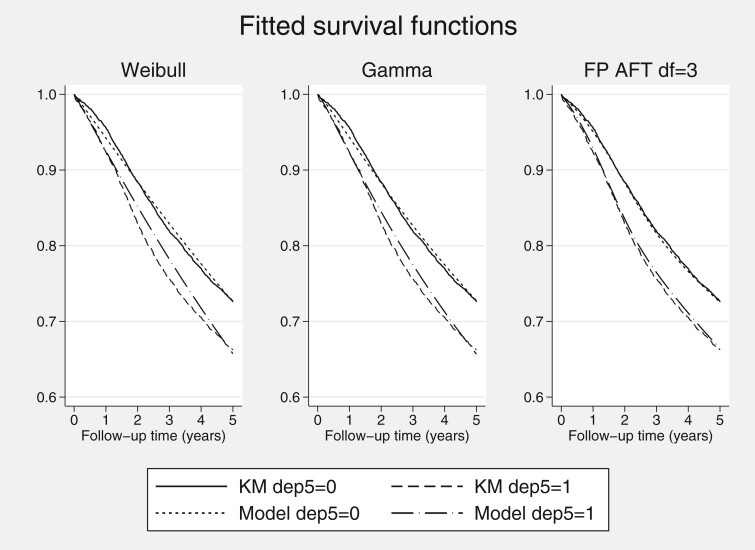
Fitted survival for each deprivation groups, for Weibull, gamma, and Flexible parametric AFT (df ${=}$ 3) models.

**Fig. 4:**
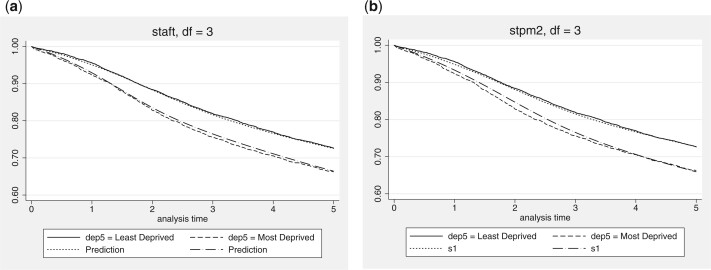
Fitted survival function for the best fitting flexible parametric models in the proportional hazards and accelerated failure time metrics. (a) Proportional hazards model. (b) Accelerated failure time model.

We illustrate the fitted AFT models in Figure 3, showing the fitted survival function for both deprivation groups (male, age = 42), for the Weibull, gamma, F and best fitting flexible AFT model, overlaid on the Kaplan–Meier estimates. We further show a comparison to the proportional hazards Royston–Parmar model in Figure 4, illustrating that the flexible AFT fits substantially better than the equivalent proportional hazards metric formulation, in this case. 

**Fig. 5:**
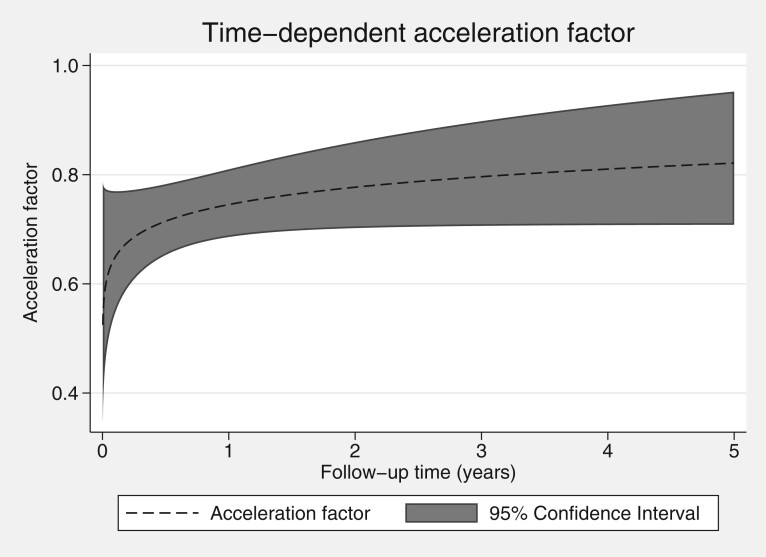
Estimated time-dependent acceleration factor for the association between deprivation status and survival.

Finally, we investigate the presence of a time-dependent acceleration factor for the effect of deprivation status on survival time. We found that 1 degree of freedom was sufficient, with the estimated time-dependent acceleration factor shown in Figure 5, indicating an initial low acceleration factor early on during follow-up with a subsequent, with a subsequent attenuation over follow-up time. 

## 6. Discussion

AFT models provide an attractive alternative to the proportional hazards framework, particularly for patients, as an acceleration factor can have a more intuitive meaning, directly increasing or decreasing survival time, rather than the event rate. Many authors have argued that AFT models are underused in applied research (Swindell, 2009; Kay and Kinnersley, 2002; Ng *and others*, 2015). Indeed, estimates have been shown to be more robust to covariate omission, compared to proportional hazards models (Lambert *and others*, 2004; Keiding *and others*, 1997). In this article we proposed a new general parametric AFT model. We focused on the use of restricted cubic splines to provide a highly flexible framework with which to capture complex, biologically plausible functions. Our model can be thought of as an AFT formulation of that proposed by Royston and Parmar (2002). Furthermore, we extended the framework to allow time-dependent acceleration factors, and illustrated with an example in breast cancer, showing how we can capture a time-dependent effect within the AFT framework. 

AFT models show considerable promise for causal inference. In particular, the log acceleration factors are collapsible for omitted covariates that are uncorrelated with the exposure of interest, whereas the proportional hazards models are sensitive to such random effects or frailties. Moreover, the proportional hazards model have a difficult causal interpretation (see (2.1)). 

We conducted a simulation study to evaluate the performance of the proposed AFT model, indicating overall good performance in a variety of complex, but plausible, settings. In our scenarios, it outperformed the Weibull, generalized gamma and generalized F models, both in terms of minimizing bias in estimates of the acceleration factor and coverage probabilities closer to the optimum 95$\%$. Furthermore, we found that model selection criteria can aid in selecting degrees of freedom, both to select a model with minimal bias, but also a model which capture the baseline to provide reliable estimates of absolute risk such as survival probabilities. The proposed flexible parametric AFT models is also highly computationally efficient. We compared our implementations to that of Pang *and others* (2021), using the example presented in their paper, finding that their provided code took approximately 26 000 s on a fast laptop, compared to approximately 1500 s using the smoothsurv package, and compared to our implementations of the above model framework, taking 0.4 s in R and 0.15 s in Stata. 

An AFT model will be most appropriate when the covariate effects are multiplicative on a time scale. This scale is intuitive for modeling life expectancy, but is more difficult to interpret in terms of competing risks. To describe this difficulty, consider dividing causes of death into two groups, where an exposure affect the causes of death with different acceleration factors. Then survival from all cause will be the product of two survival probabilities which have “ageing” at different rates for the different causes. 

In contrast, the proportional hazards models assume that the covariate effects are multiplicative on the hazards scale. This scale is more intuitive for modeling system dynamics and for competing risks, but these models have a difficult causal interpretation and they are less intuitive for the lay person. As a third model class, the additive hazards model are intuitive for effects operating as competing events and have a straightforward causal interpretation; however, they are less intuitive for interpreting effects on the same mechanistic pathway. Further comparison between alternative modelling frameworks are warranted, especially comparing model performance between proportional hazards and accelerated failure time frameworks. We are actively undertaking such comparisons in further work. 

Extensions to the framework that would be useful include incorporating penalized smoothers, random effects, to account for clustered structures and unobserved heterogeneity (Lambert *and others*, 2004; Crowther *and others*, 2014), and the extension to interval censoring. This class of AFT models is closely related to flexible parametric survival models, where the baseline time-to-event distributions are modeled in a similar manner. In general, we expect that extrapolations outside of observed data will have similar properties to the flexible parametric survival models. This is also an area for further research. 

We provide user-friendly Stata and R software packages to allow researchers to directly use the proposed model framework. For Stata, the command can be installed by typing ssc install staft. For R, the rstpm2 package on CRAN provides an aft regression function. 
